# Encoding and Storage of Information in Mechanical Metamaterials

**DOI:** 10.1002/advs.202301581

**Published:** 2023-04-21

**Authors:** Zhiqiang Meng, Hujie Yan, Mingchao Liu, Wenkai Qin, Guy M. Genin, Chang Qing Chen

**Affiliations:** ^1^ Department of Engineering Mechanics, CNMM and AML Tsinghua University Beijing 100084 P. R. China; ^2^ School of Mechanical and Aerospace Engineering Nanyang Technological University Singapore 639798 Republic of Singapore; ^3^ Mechanical Engineering and Materials Science Washington University St. Louis MO 63130 USA; ^4^ NSF Science and Technology Center for Engineering Mechanobiology St. Louis MO 63130 USA

**Keywords:** information encoding, multistep deformation, pixelated mechanical metamaterials, tunable bistability

## Abstract

Information processing using material's own properties has gained increasing interest. Mechanical metamaterials, due to their diversity of deformation modes and wide design space, can be used to realize information processing, such as computing and storage. Here a mechanical metamaterial system is demonstrated for material‐based encoding and storage of data through programmed reconfigurations of the metamaterial's structured building blocks. Sequential encoding and decoding are achieved in the three‐dimensional (3D) printed pixelated mechanical metamaterial via kirigami‐based “pixels” with programmable, temperature‐dependent bistability. The mechanical metamaterial is demonstrated via a multistep deformation of encoding messages of texts and surfaces with arrays of binary data, and then decoding them by applying a predetermined stretching and heating regimen to sequentially retrieve layers of stored information and display them on its surface. This approach serves as a general framework to enable the encoding and storage of data with mechanical metamaterials.

## Graphical abstract

The paper reports a pixelated mechanical metamaterial that can be 3D printed with multiple layers of information within its structure. The mechanical metamaterial can reveal the stored multiple messages sequentially on a surface of the material over the course of a series of physical manipulations such as specified heating and stretching.

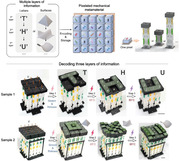

## 1 Introduction

Mechanical metamaterials, consisting of carefully structured building blocks, have properties either not available from or surpass their constituent materials.^[^ ^1^, ^2^ ^]^ Their stiffness and strength‐to‐weight ratio,^[^ ^3^, ^4^ ^]^ Poisson's ratio,^[^ ^5^, ^6^, ^7^ ^]^ energy absorption capacity,^[^ ^8^, ^9^, ^10^ ^]^ fracture resistance,^[^ ^11^ ^]^ and shape transformation^[^ ^12^, ^13^, ^14^, ^15^, ^16^ ^]^ can be tuned to match or exceed those found in conventional materials. When combined with stimuli‐responsive materials, mechanical metamaterials can provide new opportunities of developing novel functionalities that react, deploy, and evolve in specific environments or conditions directly.^[^ ^17^, ^18^, ^19^, ^20^, ^21^, ^22^, ^23^, ^24^, ^25^, ^26^ ^]^ For example, materials can hide and reveal information through liquid‐induced structural transformation^[^ ^19^ ^]^ or mechanical compression;^[^ ^13^, ^27^ ^]^ 3D microlattices can transform into other patterns through an electrochemical reaction;^[^ ^20^ ^]^ magnetic field,^[^ ^21^, ^22^ ^]^ electric current,^[^ ^23^, ^24^ ^]^ and heat^[^ ^25^, ^26^ ^]^ can be applied for reconfiguring metamaterials into different geometries to adapt to the surrounding environment.

Information processing capability^[^ ^28^, ^29^, ^30^, ^31^ ^]^ has recently been shown to be a possible additional material property, including mechanical computing,^[^ ^32^, ^33^, ^34^, ^35^ ^]^ memory formation,^[^ ^36^, ^37^, ^38^ ^]^ and learning.^[^ ^39^, ^40^ ^]^ Storing information through the material itself is an issue of fundamental importance, and efforts have been devoted to exploring accessible approaches to achieve information encoding and storage. Optical encryption,^[^ ^41^, ^42^, ^43^ ^]^ for example, can encode and decode target information through optical metasurfaces by exploiting the abundant degrees of freedom of light, possessing high accuracy and versatility. There are several studies about encoding information with mechanical metamaterials through external stimuli.^[^ ^13^, ^19^, ^44^, ^45^ ^]^ However, high density information storage with mechanical metamaterials remains challenging. For example, only one stored information can be decoded at the phase boundaries by treating the liquid‐crystalline polymer microstructure with different solvent systems;^[^ ^19^ ^]^ at most two letters of alphabet can be encoded into a shape‐morphing micromachine due to its single‐layer structure and limited interconnectivity between unit cells.^[^ ^44^ ^]^

**Figure 1:**
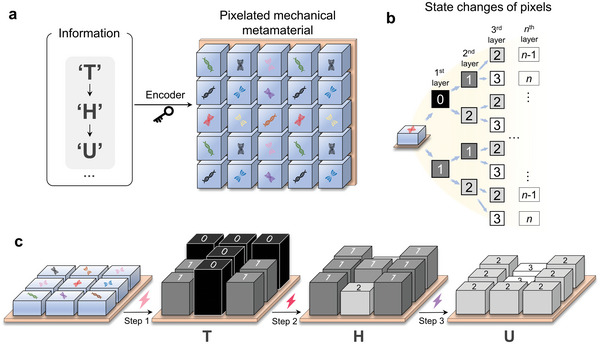
Schematic of encoding and storage of multiple layers of information using a pixelated mechanical metamaterial. a) Three layers of information (i.e., the first is “T”, the second is “H”, and the third is “U”) are encoded and stored into one pixelated mechanical metamaterial that is composed of different pixels. b) State changes of pixels to encode *n* layers of information. Two states in each layer of information, i.e., “0” and “1” states in the first layer; “1” and “2” states in the second layer; “*n*‐1” and “*n*” states in the *n*th layer. c) The pixelated mechanical metamaterial is decoded to obtain “T”, “H”, and “U” in sequence by applying stimuli.

Pixelated mechanical metamaterials^[^ ^46^, ^47^, ^48^, ^49^ ^]^ that can decouple the deformation constraints between interconnected building blocks and thus greatly increases the deformability and programmability of materials have been proposed. We developed a pixelated mechanical metamaterial to enable encoding and storage of denser information within the physical structure of a material system (**Figure** **1**a). Temperature‐responsive kirigami‐based multimaterial pixels were assembled to form the pixelated mechanical metamaterials. Multiple layers of encoded information (e.g., letters or surfaces) could be decoded sequentially by stretching and varying the ambient temperature. A general design framework for designing the metamaterials was obtained and applied to a Matlab‐based tool, thus enabling multiple desired messages to be encoded in the material.

## 2 Results

### 2.1 Design Framework for Information Encoding and Storage

A schematic diagram of the pixelated mechanical metamaterial with multiple layers of information is shown in Figure 1a. The three layers of information (Figure 1a left, “T”, “H”, and “U”) can be sequentially encoded and stored in the mechanical metamaterial using an encoder that specifies how each pixel changes state. The symbols of gene on pixels (Figure 1a right) represents how the state of each pixel changes. To enable independent storage of each layer of information, physical realization requires that the pixels fulfill the state changes shown in the tree diagram of Figure 1b (see Section S3.1. in the Supporting Information for the construction of the tree diagrams), in which each layer of information is represented by two states (e.g., states “0” and “1” for the first layer; states “1” and “2” for the second layer, etc.). Additional stable states in each layer (e.g., states “0”, “1”, and “2” for the first layer, etc., see Figure 5a and Figure S9, Supporting Information) can encode richer information. The number of layers of information is limited only by the number of stable states in the pixels. To experimentally realize the pixelated mechanical metamaterials with multiple layers of information, each constituent pixel needs to satisfy the following requirements: first, pixels must be able to achieve multiple stable states; second, pixels must be able to switch between different states by responding to external stimuli; third, pixels must not interact with their neighboring pixels. For this work, different heights of pixels generated by external stimuli were used to represent various states, thereby presenting stored information. The schematic of the decoding process (Figure 1c) shows a sequential expression of the texts (i.e., “T”→“H”→“U”) by sequentially applying different external stimuli (i.e., multistep loading) which are marked by colored lighting symbols.

### 2.2 Construction of Pixelated Metamaterial

**Figure 2:**
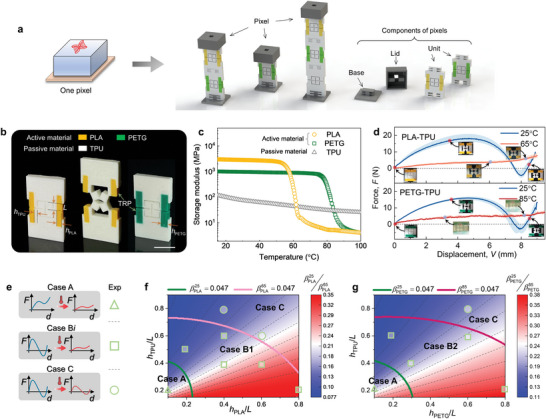
Design and mechanical responses of pixels. a) Components of a pixel, including kirigami‐based units, base, and lids. b) Photograph of bimaterial kirigami‐based units. c) Storage modulus versus temperature for the passive material (TPU) and active materials (PLA and PETG). d) Experimental force–displacement curves of two units at different temperatures. Top, the unit cell made of PLA and TPU is stretched at 25 and 65 °C. Bottom, the unit cell made of PETG and TPU is stretched at 25 and 85 °C. Snapshots of the deformation process are labeled. E–g) Effects of $h_{\mathrm{TPU}}/L$, $h_{\mathrm{PLA}}/L$, and $h_{\mathrm{PETG}}/L$ on the tunable bistability by varying temperature. e) Cases A, B*i*, and C represent monostability, tunable bistability, and bistability, respectively. Phase diagrams for the unit cells made of f) PLA and TPU, g) PETG and TPU. See Video S1 in the Supporting Information. Scale bars, 1 cm.

In the implementation shown here, the pixels we presented here were composed of serial‐connected kirigami‐based units, lids, and bases (**Figure** **2**a, see Video S1, Supporting Information). 3D printed multimaterial kirigami‐based units were subject to stretching and varying temperatures (Figure 2b–g). These temperature‐responsive units were comprised of materials whose elastic moduli change differently with temperature. As shown by dynamic mechanical analysis (Figure 2c, see Experimental Section “Characterizations of materials”), polylactic acid (PLA) and polyethylene terephthalate (PETG) have moduli that drop significantly when heated above their glass transition temperatures, i.e., T_1_ = 65 °C for PLA and T_2_ = 85 °C for PETG. In contrast, the modulus of thermoplastic polyurethane (TPU) is relatively insensitive to temperature change. It should be pointed out that the rationale for selecting the active materials is that the contrast of their stiffness to the passive material can be varied significantly by heating or cooling. Such a feature is vital to achieving the switchable bistability that is desired in this study. As to TPU, it was chosen for its good flexibility, durability and ability to withstand repeated deformation cycles without degradation.

Inspired by kirigami paper cutting, kirigami‐based units (Figure 2b) were printed via bi‐material fused deposition modeling of passive (TPU, white) and active materials (PLA, yellow, or PETG, green; see Experimental Section and Section S1.1., Supporting Information). The units contained four square plates (edge length *L*), each linked by a cantilever that is a composite of temperature‐responsive parts (TRPs, width *h*_PLA_ for PLA or *h*_PETG_ for PETG) and passive elements (width *h*_TPU_ for TPU). Transformation proceeded by stretching the unit until it adopted a second stable state (Figure 2b, middle). Experimentally measured force‐displacement curves (Figure 2d) of the PLA‐TPU and PETG‐TPU units under axial stretching revealed bistability at 25 °C, meaning that they lock into a second stable state after unloading. The bistability is mainly due to the strong constraint exerted on the square plates by the composite cantilevers. This state unlocked when bistability disappeared above the glass transition temperature for PLA or PETG; reversible bistable composite cantilevers softened relative to other parts, the constraint locking the squares into the deformed state weakened and the stretched units returned to their original states (see Video S1, Supporting Information).

The stability of bimaterial kirigami‐based units could be predicted as functions of $h_{\mathrm{TPU}}/L$ and $h_{\mathrm{PLA}}/L$ ($h_{\mathrm{PETG}}/L$), with three phases appearing in the phase diagram (Figure 2e–g; see Section S2, Supporting Information, for theoretical model): a monostable phase (Case A); one of two tunable bistable phases realized by heating (Case B1 for PLA‐ and B2 for PETG‐based bimaterial kirigami units); and a bistable phase (Case C). The model was verified against experimental measurements (symbols in Figure 2f,g), then adopted for programming and design of subsequent kirigami‐based units. Four representative units were chosen for further study based on these phase diagrams: Cases A and C, TPU‐printed with *h*_TPU_ = 2 and 5 mm, respectively; Case B1, PLA‐TPU‐printed with *h*_PLA_ = 4mm and *h*_TPU_ = 1mm; and Case B2, PETG‐TPU‐printed with *h*_PETG_ = 4mm and *h*_TPU_ = 1mm (see Figure S5, Supporting Information).

### 2.3 Encoding and Storage of One‐Layer Information

**Figure 3:**
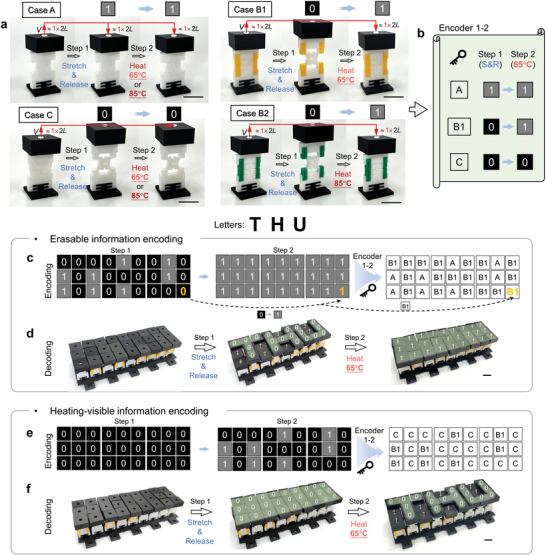
Encoding and storage of one layer of information in a pixelated mechanical metamaterial. a) Definition of “0” state and “1” state for the one‐layer pixel by two‐step operation, i.e., the first step, stretching and releasing (S&R), the second, heating. Case A and Case C are “1” → “1” and “0” → “0”. Case B1 and Case B2 are all correspond to “0” → “1”. b) Encoder 1–2 is determined by the status change of Cases A, B1, and C. c,d) Erasable information encoding. c) Encode the letters “THU” in Step 1 and erases it in Step 2. d) The stored information can be displayed by stretching and releasing (Step 1), and hid by heating (Step 2). e,f) Heating‐visible information encoding. e) Encode the letters “THU” in Step 2 and use the first step as a jamming message. f) The stored information can be displayed in Step 2. See Video S2 in the Supporting Information. Scale bars, 2 cm.

As for the encoding and storage of one layer of information in a pixelated mechanical metamaterial, the state changes of pixels are shown in **Figure** **3**a,b. Transitions between the “0” and “1” states occurred through a two‐step operation. As shown in Figure 3a, a pixel was elongated to 2*L* and then released (Step 1), and heated to 65 or 85 °C for returning to its original state (Step 2). For instance, a Case B1 pixel begins in the “1” state at room temperature before being stretched by 2*L* and released (Step 1). The pixel retains this elongated (“0”) state due to its bistability at 25 °C. Upon heating to 65 °C (Step 2), the pixel loses bistability and snaps back to the “1” state. Case A pixels stay in the “1” state under these conditions due to the monostability, and Case C pixels stay in the “0” state. Since heat transfer within materials requires a certain amount of time, it is necessary for the heating process to last for more than 70 s to ensure the material fully heated (see Figure 6c). The encoding approach (Encoder 1–2) is thus obtained for combinations of Case A, B1, and C pixels, with “Encoder *m*‐*r*” representing encoding and storage for *m* layers of information, each containing *r* states. For example, the Case A, B1, and C pixels in Figure 3b are denoted by Encoder 1–2. This can also be obtained by replacing the Case B1 pixel with a Case B2 pixel for an Encoder 1–2 that returns to state “1” after heating to 85 °C.

To illustrate the framework (Figure 1a), we first used Encoder 1–2 (Figure 3b) to encode the text “THU” into a pixelated mechanical metamaterial via two methods, i.e., erasable information encoding (Figure 3c,d) and heating‐visible information encoding (Figure 3e,f). There are three steps. First, the three letters were discretized onto a 3 × 9 pixel array and used the “0” state to express this information in Step 1, while the rest was filled with the “1” state (Figure 3c, left). The state of all pixels was “1” in Step 2 to enable the erasure or hiding of the information (Figure 3c, middle). Second, by combining with the Encoder 1–2, pixel arrays were obtained to generate the corresponding design of the pixels of the pixelated mechanical metamaterials (Figure 3c, right). For example, because the pixel in the lower right of the pixel map (yellow pixel, Figure 3c) must change from “0” to “1”, a Case B1 kirigami‐based unit was selected for that pixel according to Encoder 1–2. Third, the mechanical metamaterial stored information “THU” could be 3D printed and assembled (Figure 3d, left) according to the pixel design. The stored information in the mechanical metamaterial was displayed and erased through two operations (Figure 3d, see Video S2, Supporting Information). First, all pixels were stretched and released to display the stored information through the distribution of “0” and “1” states (“THU”, green in Figure 3d, middle). Second, upon heating the pixelated mechanical metamaterial to 65 °C in a water bath, the information could be erased by shifting all pixels back to the “1” state (Figure 3d, right). We refer to this approach as erasable information encoding.

Even in this simple, single layer demonstration, other permutations are possible. For example, the data could be encoded and stored in the second step (Figure 3e, middle) while the first pixel map was filled with “0” state pixels. Then, the designed pixelated mechanical metamaterials could be decoded the data by heating (Step 2) because no data was revealed in response to stretching and releasing (Figure 3f). Therefore, this approach shown in Figure 3e,f is capable of decoding through heating. The two examples shown in Figure 3c–f demonstrate the use of PLA‐based pixels to encode and decode one layer of information. Similarly, PETG‐based pixels can be used to realize these functions. The main difference between the two is that PETG‐based units require a higher heating temperature (i.e., 85 °C) to achieve the transition of the units from bistability to monostability.

### 2.4 Encoding and Storage of Multiple Layers of Information

In the first demonstration, the pixelated mechanical metamaterial stored only one layer of information, a limitation arising from having no pixel that could change state from “1” to “0” (see Figures S9 and S10, Supporting Information). To enable two layers of information to be stored independently in the same mechanical metamaterial, we increased the number of kirigami‐based units in the pixels. An example is a pixel constructed with a combination of two units within Cases A, B1, and C, generating the extended encoding approach (Encoder 2–2) for two layers of information (see Figure S11 and Video S3 in the Supporting Information for details).

**Figure 4:**
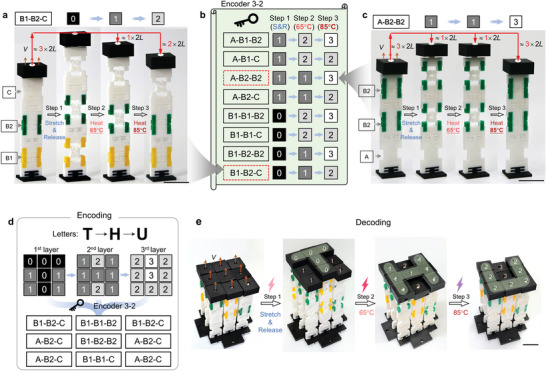
Encoding and storage of three layers of information in a pixelated mechanical metamaterial. A–c) Definition of Encoder 3–2 using three‐unit pixels. a) A pixel, composed of Cases B1, B2, and C (B1‐B2‐C), achieves the state change “0”→“1” →“2” through a three‐step operation, that is, stretching and releasing (Step 1), heating to 65 °C (Step 2), and heating further to 85 °C (Step 3). c) The pixel (A‐B2‐B2) realizes “1” →“1” →“3”. b) Encoder 3–2 with two states for each layer. d) Pixelate the letters (“T”, “H”, and “U”) into 3×3 array and encode them into a pixelated mechanical metamaterial through Encoder 3–2. E) The pixelated mechanical metamaterial decoded to obtain “T”, “H”, and “U” in sequence. See Video S4 in the Supporting Information. Scale bars, 2 cm.

A pixelated mechanical metamaterial with three layers of binary information was similarly obtained using pixels composed of three units connected in series (**Figure** **4**). For illustration, a pixel composed of Cases B1, B2, and C (B1‐B2‐C) was developed (Figure 4a). The pixel was first fully stretched by a displacement of 6*L* and remained in this “0” state after release due to the bistability in all constituent units (Step 1). Upon heating to 65 °C (Step 2), the Case B1 unit switched from bistable to monostable and shrank back a distance 2*L* to move the lid to the “1” state. Upon subsequent heating to 85 °C (Step 3), the Case B2 unit became monostable and the lid shifted another displacement of 2*L* to the “2” state. Therefore, a B1‐B2‐C pixel can change the state from “0” to “1” to “2” in a three‐step operation (Figure 4a). Similarly, an A‐B2‐B2 pixel (Figure 4c) can change the state from “1” to “1” to “3” by applying the same operation. Following the tree diagram (Figure 1a), the encoding approach (Encoder 3–2) of the pixelated mechanical metamaterials with three layers of information and two states for each layer was obtained by combining three of Cases A, B1, B2, and C (Figure 4b).

Then we wanted to encode “THU” so that information emerges sequentially from a single 3 × 3 pixel array (i.e., “T”, then “H”, then “U”) rather than all at once on a larger array as in the previous example. First, the three letters were discretized onto three 3 × 3 pixel arrays and used “0”, “1”, “2”, and “3” states to express the information in the first, second, and third layer respectively (Figure 4d). Second, by combining with Encoder 3–2, pixel arrays were obtained to generate the corresponding design of the pixels of the pixelated mechanical metamaterials (Figure 4d, bottom). Third, the pixelated mechanical metamaterial with three layers of information was fabricated and assembled according to the pixel design (Figure 4e, left). The pixelated mechanical metamaterial was decoded into text through three steps (Figure 4e, see Video S4, Supporting Information): first, stretching and releasing all pixels, which reveals the letter “T”; second, heating at 65 °C, which reveals the letter “H”; and third, heating at 85 °C, which reveals the letter “U”.

Extending the foregoing analysis, one can realize pixelated mechanical metamaterials storing *n* layers of binary information by using *n* − 1 units with different thresholds of tunable bistability. For example, if a third active material with a glass transition temperature of 95 °C is used, Case B3 can be obtained by bi‐material printing it with TPU and to identify the encoding approach of four layers of information by applying the tree diagram of Figure 1b. Additional units are possible that transform in response to other external stimuli such as light, electric fields, chemical treatment, or magnetic fields.

**Figure 5:**
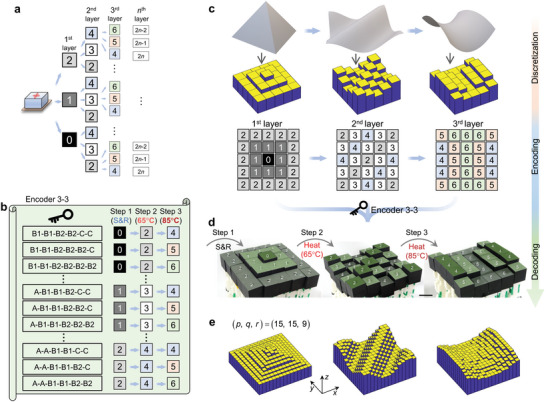
Encoding and storage of 3D surface information. a) State changes of pixels to encode *n* layers of information. Three states in each layer of information, i.e., “0”, “1”, and “2” states in the first layer; “2”, “3”, and “4” states in the second layer; “2*n*‐2”, “2*n*‐1”, and “2*n*” states in the *n*th layer. b) Encoder 3–3 with three states for each step. c,d) Encoding and storage of 3D surface information. c) Discretization of three surfaces (pyramid, wave, and hyperbolic paraboloid) into three pixel maps. d) The pixelated mechanical metamaterial is decoded to obtain three surfaces in sequence. e) High‐resolution discretized surfaces with (*p*, *q*, *r*) = (15, 15, 9). See Video S5 in the Supporting Information. Scale bars, 2 cm.

After these demonstrations of binary data encoding and storage (Figures 3 and 4), we adapted the framework to enable the digital discretization of data by adding stable states in each layer (**Figure** **5**a; Figure S9b,c, Supporting Information). In a demonstration of the encoding and storage of three‐dimensional (3D) surfaces, each layer had three states (i.e., states “0”, “1”, and “2” in the first layer; states “2”, “3”, and “4” in the second, etc.), and the encoding approach (Encoder 3–3) contained three layers of information (Figure 5b, see Figure S19 in the Supporting Information for its full version). To demonstrate discretization, we encoded and stored height maps of three different surfaces – a pyramid, a wave, and a hyperbolic paraboloid – into a pixelated mechanical metamaterial and displayed them sequentially (Figure 5c,d). In this example, the three surfaces were discretized onto three 5 × 5 pixel arrays using three states that represent the heights of pixels on the surfaces (Figure 5c, see Section S3.5. in the Supporting Information for discretization method), and Encoder 3–3 was used on the three pixel maps to encode data into the pixelated mechanical metamaterial (Figure S15, Supporting Information). The decoding process occurred by stretching and releasing (Step 1), heating at 65 °C (Step 2), and heating at 85 °C (Step 3), which displayed the three surfaces in sequence (Figure 5d, see Video S5, Supporting Information). To discretize surfaces more accurately, we increased the spatial resolution of pixel maps by increasing the number of pixels in the *x*‐ and *y*‐directions (denoted by *p* and *q*), and increased the number of states in each layer of information denoted by *r*. The three surfaces could be discretized more precisely with high resolution (i.e.,*p* = *q* = 15, *r* = 9) and encoded to a pixelated mechanical metamaterial in theory (Figure 5e, see Section S3.6. in the Supporting Information for details).

A computational “Encoder” tool applies these principles to design pixels for pixelated mechanical metamaterials (see the Experimental Section “Matlab‐based encoder” and Software S1 in the Supporting Information). The graphical user interface (GUI) of the Encoder allows the input of two types of information, i.e., surfaces or words. For surfaces, either a mathematical expression or an STL format graphic file can be used as an input. For letters and numbers, a virtual keyboard can be used to enter any text. After entering these messages, three typical resolutions (i.e., low, medium, and high) are available. Based on the above inputs, Encoder then generates pixel arrays of the pixelated mechanical metamaterials. The Matlab‐based tool is available for download (Software S1, Supporting Information) and a video showing how to use this Encoder is provided in Video S6 in the Supporting Information.

### 2.5 Tailored Temporal Responses by Thermal Management

**Figure 6:**
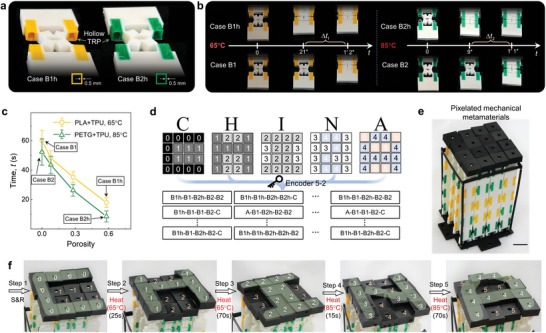
Tailored temporal responses by thermal management. a) Photograph of bimaterial kirigami‐based units with hollow TRPs. b) Comparison of the recovery time of stretched units with hollow and solid TRPs at different temperatures (65 and 85 °C). c) The dependence of the recovery time on porosity. d) Encoding the letters (“C”, “H”, “I”, “N”, and “A”) to a pixelated mechanical metamaterial with 4×4 pixels. e) The pixelated mechanical metamaterials with five layers of information. f) Decoding process of mechanical metamaterials, acquiring five messages in turn, i.e., “C”→ “H”→ “I”→ “N”→ “A”. See Video S7 in the Supporting Information. Scale bars, 2 cm.

The temporal responses of pixelated mechanical metamaterials could be tailored to give rise to an expanded design space (**Figure** **6**). Besides the regulation by different temperatures, the response time was considered as an extra manipulation factor. Different from the units with solid TRPs (Figure 2b), the bimaterial printed units of Case B1h and Case B2h with hollow TRPs were developed and denoted by “h” in Figure 6a. In Figure 6b, when Cases B1h and B1 at the second stable state were heated at 65 °C, there was a time gap (Δ*t*_1_) in their occurrences of snap‐back, which means Case B1h could return to the initial unstretched state faster than Case B1. Similarly, Case B2h could respond faster than Case B2 (Figure 6b right). The reason is that heat can transfer faster in the hollow TRPs, resulting in faster softening. We conducted experiments on units with various porosities of TRP (see Figure S18, Supporting Information) by immersing them in the water of constant temperature (e.g., 65 and 85 °C) and recording their recovery time (Figure 6c). Porosity is defined as the ratio of the hollow region to the total cross‐sectional area of TRP. Note that no material is filled into the hollow. Four specimens for each porosity were tested. The experimental data in Figure 6C revealed that there was an apparent time gap between Cases B1h and B1, as well as B2h and B2. To enable encoding and storage of five layers of information with time‐dependent morphing, pixels were therefore constructed by combining five units (Cases A, B1h, B1, B2h, B2, and C). The encoding approach for these pixels required algorithms that accounted for temporal responses. The encoding approach (Encoder 5–2, see Figure S20, Supporting Information) would involve five steps: Step 1, stretch and release; Step 2, heat at 65 °C for 25 s, during which time Case B1h snaps back but not Case B1; Step 3, heat at 65 °C for 70 s or longer, ensuring that Case B1 snaps back; Step 4, heat at 85 °C for 15 s, during which time Case B2h snaps back; and Step 5, heat at 85 °C for 70 s or longer, ensuring that Case B2 snaps back (see Figure S20 in the Supporting Information for its full version). Applying this, five layers of information (i.e., “C”, “H”, “I”, “N”, and “A”) was encoded into a 3D printed pixelated mechanical metamaterials (Figure 6d,e), and then decoded one letter at a time (i.e., “C”→“H”→“I”→“N”→’A’, Figure 6f, see Video S7, Supporting Information). The time‐dependent behaviors of the structured materials can further enrich the diversity of the method of information encoding and storage in addition to the choice of different materials.

## 3 Conclusion

The proposed pixelated mechanical metamaterials enabled encoding and storage of a range of text and surfaces via temperature‐responsive kirigami‐based multimaterial pixels arranged over multiple layers of a metamaterial. Although at present these demonstrations are not yet practical for large‐scale data storage, they do provide a metamaterial perspective on information storage and material design. With advances in 3D printing at the submicrometer scale^[^ ^50^ ^]^ and using multiple materials,^[^ ^51^ ^]^ the miniaturization needed for higher storage capacity may soon be within reach owing to the scale‐independence of the underlying physical mechanisms. The design strategy suggests pathways to creating additional stimulus‐responsive materials that can achieve multifunction. More broadly, the work demonstrates that material with information encoding and storage is possible within the context of structured metamaterials.

## 4 Experimental Section

### Characterization of Materials

To measure the stress–strain curves of TPU, PLA, and PETG at room temperature (25 °C), uniaxial tensile tests of dog‐bone‐shaped specimens were performed. A material testing machine (Zwick Z005) was equipped with a 5 kN load cell. Specimens were fully clamped at both ends and stretched at a strain rate of 0.2 min^−1^ at 25 °C. As for the stress–strain responses at higher temperatures (65 and 85 °C), tensile tests of TPU, PLA, and PETG were conducted using a uniaxial testing machine (SANS CMT5105S) that was equipped with a 50 N load cell and a thermal chamber. The temperature was set to be 65 or 85 °C. The experimentally measured data can be seen in Section S1.1. in the Supporting Information. The dynamic thermomechanical properties were obtained by dynamic mechanical analysis, using a TA Instruments RSA3 dynamic mechanical analyzer. The dimensions of the PLA and TPU samples were 25×8×2 mm. The samples oscillated at 1 Hz to 0.02% strain. The temperature was ramped from 15 to 100 °C at a ramp rate of 0.5 °C per minute. The temperature‐dependent storage modulus and loss factor are shown in Figure 2b.

### Bimaterial 3D Printing

Single‐ and bimaterial units were printed using the fused deposition modeling with a nozzle diameter of 0.4 mm (Ultimaker 3), with the height of the first layer set to be 0.27 mm and the height of all subsequent layers being 0.1 mm with an infill density of 100%. The printing speed was 25 mm s^−1^ with a retraction distance of 3 mm. The nozzle temperature was set to be 223 °C for TPU, 200 °C for PLA, and 235 °C for PETG. The printed unit is shown in Figure S3b in the Supporting Information. To prevent debonding on the interfaces between PLA and TPU or PETG and TPU, it was necessary to allow them to overlap slightly. In the overlapped regime, PLA and TPU were alternately stacked (Figure S3c, Supporting Information) to achieve strong bonding.

### Assembly of Pixels

As shown in Figure 2a and Figure S4 (Supporting Information), units were combined, one base, and one lid to construct a pixel. Herein, considering the influence of high temperatures, the lid and base were made of high temperature resistant Nylon (Weilai 7500) using 3D printing. This material has a high heat deflection temperature (175 °C) so that the lid and base of the pixel still have adequate stiffness at 65 and 85 °C. The units were designed into a tenon‐and‐mortise structure so that they could be assembled flexibly to construct a pixel.

### Measured Force–Displacement Curves of Units

To perform tensile tests of the units at 65 and 85 °C, a water bath tensile test platform was built consisting of a metal collet, a digital water bath, and a uniaxial testing machine (Zwick Z005). The digital water bath could heat water to control the temperature between 25 and 100 °C. The metal collet was immersed in the digital water bath. The bottom end of the tested unit was fixed by the collet's weight. The upper end was affixed to the uniaxial testing machine. During stretching, tested units were immersed in water continuously. The digital water bath was also used for heating tests of the ciphermaterials. Uniaxial tensile test data of four different units (i.e., Cases A, B1, B2, C) at different temperatures are shown in Figure S5 in the Supporting Information. Four specimens of each unit were tested at higher temperatures and their measured force–displacement curves are shown as the blue and red areas in Figure S5e–h (Supporting Information), in which the solid lines denote the average of the experimental results. The experimentally measured force–displacement curves show that Cases B1 and B2 transform from bistability to monostability upon heating, while Cases A and C maintain their original monostability even if heated.

### Matlab‐Based Encoder

A MATLAB graphical user interface (GUI) was designed to determine the structure of the metamaterial with the desired information. Its working process is given in Figure S12 in the Supporting Information. First, users choose from the tab options to decide either surfaces or letters are to be encoded. For the former, the second step is to input a specific shape of the surfaces in sequence. For the sake of versatility, both mathematical expressions and 3D surface models in STL format are acceptable. For the encoding and storage of words, letters and numbers can be typed through a virtual keyboard. After clicking the encoding button, a dialog window prompts user to choose a resolution for discretization. There are three resolution choices in the *x*–*y* plane and the *z*‐direction, that is, low resolution with (*p*, *q*) = (5, 5) and *r* = 3, medium resolution with (*p*, *q*) = (10, 10) and *r* = 6, and high resolution with (*p*, *q*) = (15, 15) and *r* = 9. Following this step, Encoder discretizes the surfaces into pixelated surfaces via pixels of different heights and realizes the visualization of input information. The discretization method is discussed in Section 3.5. in the Supporting Information in detail. Finally, the design of the ciphermaterial is displayed in another popup window. When the tool is launched, a screen will request to specify the encoding of surfaces or words.

## Conflict of Interest

The authors declare no conflict of interest.

## Author Contributions

Z.M. and C.Q.C. proposed and designed the research; Z.M. and C.Q.C. designed models and interpreted the results; Z.M., H.Y., M.L., and W.Q. performed the experiments and simulations; Z.M., M.L., H.Y., W.Q., G.M.G., and C.Q.C. wrote and edited the paper.

## Supporting information

Supporting Information

Supplemental Video 1

Supplemental Video 2

Supplemental Video 3

Supplemental Video 4

Supplemental Video 5

Supplemental Video 6

Supplemental Movie 7

## Data Availability

The data that support the findings of this study are available from the corresponding author upon reasonable request.
