# Supplementary material for: Encoding and Storage of Information in Mechanical Metamaterials
Source: Adv Sci (Weinh). 2023 Apr 21;10(20):2301581. doi: 10.1002/advs.202301581 (PMC10369242; doi:10.1002/advs.202301581)
Supplement: Supplementary file 1 — Supporting Information [file ADVS-10-2301581-s002.pdf]

ADVANCED SCIENCE
Open Access

Supporting Information

for *Adv. Sci.*, DOI 10.1002/advs.202301581

Encoding and Storage of Information in Mechanical Metamaterials

*Zhiqiang Meng, Hujie Yan, Mingchao Liu, Wenkai Qin, Guy M. Genin and Chang Qing Chen**

Supporting Information for

Encoding and storage of information in mechanical metamaterials

*Zhiqiang Meng, Hujie Yan, Mingchao Liu, Wenkai Qin, Guy M. Genin, and Chang Qing Chen**

This Supplementary Information document includes:

1. Materials and methods
2. Theoretical model
3. Supplemental results & figures
4. Captions for Videos

Other Supplementary Material for this manuscript includes the following:

Videos S1 to S7 (.mp4)

1. Materials and methods **3**

1.1 Characterizations of materials 3

1.2 Methods 5

1.2.1 Bi-material 3D printing 5

1.2.2 Assembly of pixels 5

1.2.3 Measured force-displacement curves of units 6

2. Theoretical model **7**

2.1 Mechanical model of bi-material kirigami-based units 7

2.2 Determination of stiffness K and K_θ 8

2.3 Stability phase diagrams of the units 10

3. Supplemental results & figures **13**

3.1 Construction of tree diagram 13

3.2 Finite element method (FEM) simulation 15

3.3 Encoding and storage of two layers of information 15

3.4 Matlab-based Encoder 17

3.5 Discretization method 17

3.6 Encoding of surfaces with different resolutions 20

3.7 Kirigami-based bimaterial units with different TRPs 23

3.8 Additional encoding approach 24

4. Captions for Videos **25**

5. References **26**

1. Materials and methods

1.1 Characterizations of materials

To measure the stress-strain curves of TPU, PLA, and PETG at room temperature (25°C), uniaxial tensile tests of dog-bone-shaped specimens are performed, as shown in Figure S1. The material testing machine (Zwick Z005) is equipped with a 5 KN load cell. Specimens shown in Figure S1a are fully clamped at both ends and stretched at a strain rate of 0.2 min^{-1} at 25°C.

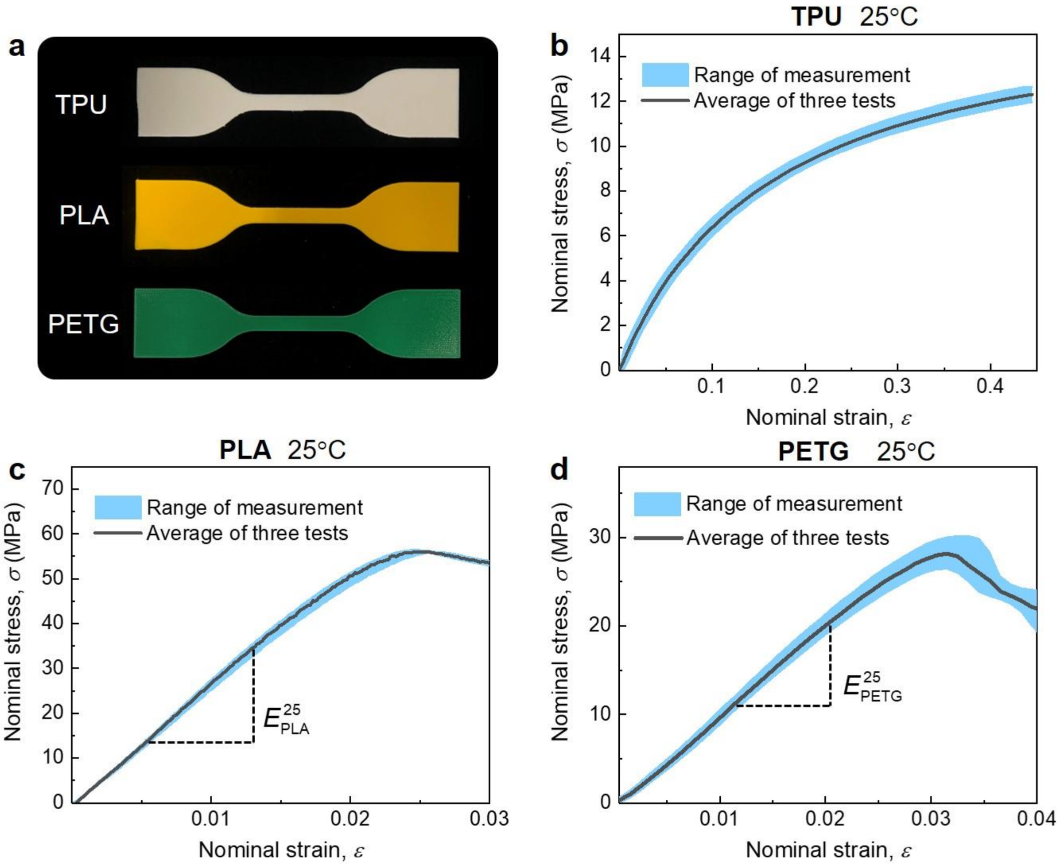

Figure S1. Measured uniaxial nominal stress-strain curves of TPU, PLA, and PETG at 25°C. a) Dog-bone shaped plate specimens. b-d) The corresponding stress-strain curves.

The measured stress-strain curves in Figure S1b to d show that TPU can be modeled by the Mooney-Rivlin hyperelastic model while PLA and PETG are approximately elastoplastic. Their corresponding material parameters can be identified as follows: for TPU, $C_{10} = -10.85$ MPa and $C_{01} = 23.98$ MPa, with Young's modulus given by $E^{25}_{\mathrm{TPU}} = 6(C_{10} + C_{01}) = 78.4$ MPa; for PLA,

Young's modulus $E_{\text{PLA}}^{25} = 2.34$ GPa, yield strength $\sigma_y = 55.6$ MPa and yield strain $\varepsilon_y = 0.024$; for PETG, $E_{\text{PETG}}^{25} = 1045$ MPa, $\sigma_y = 27.5$ MPa, and $\varepsilon_y = 0.032$.

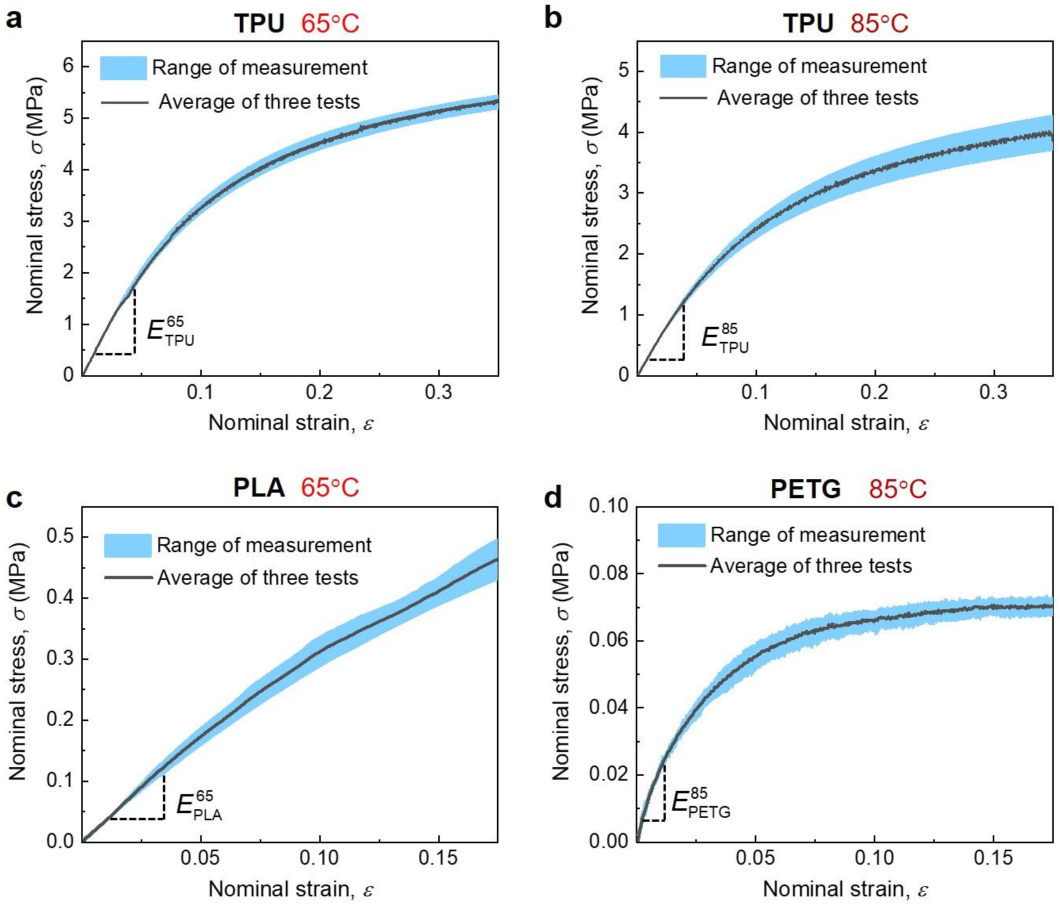

Figure S2. Measured nominal stress-strain responses of PLA and TPU at temperatures of 65°C and 85°C.

As for the stress-strain responses at higher temperatures (65°C and 85°C), tensile tests of TPU, PLA, and PETG are conducted using a uniaxial testing machine (SANS CMT5105S) that is equipped with a 50 N load cell and a thermal chamber. The temperature is set as 65°C and 85°C. The corresponding measured stress-strain curves are shown in Figure S2. The Young's moduli are inferred from the initial slope of the uniaxial stress-strain curves in the linear regime, giving $E_{\text{TPU}}^{65} = 45$ MPa, $E_{\text{TPU}}^{85} = 40$ MPa, $E_{\text{PLA}}^{65} = 5.5$ MPa, and $E_{\text{PETG}}^{85} = 2.9$ MPa, where superscripts denote the corresponding temperature.

1.2 Methods

1.2.1 Bi-material 3D printing

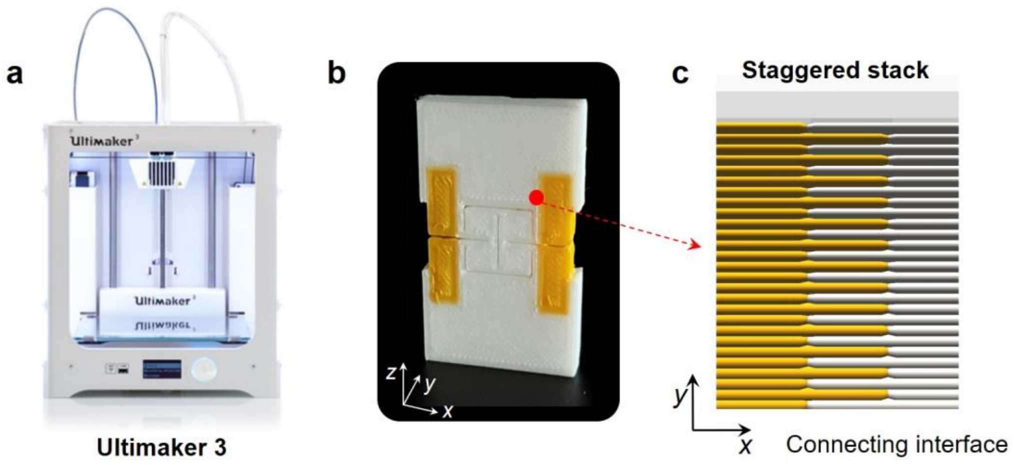

Figure S3. Fabrication of bi-material kirigami-based units. a) Bi-material printer (Ultimaker 3). b) Bi-material unit; c) Connecting interface between PLA (yellow) and TUP (white).

1.2.2 Assembly of pixels

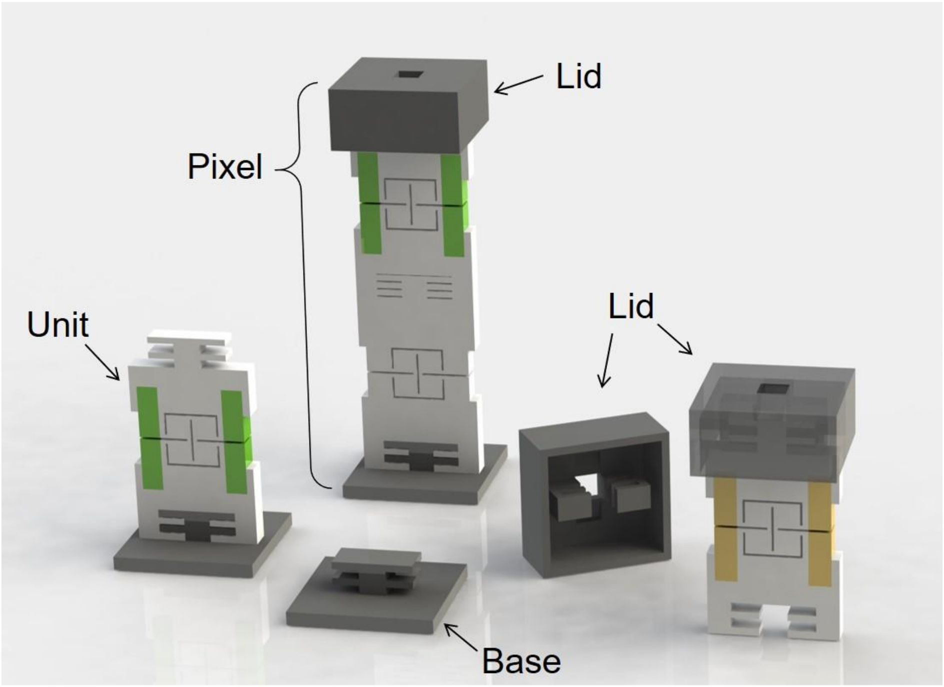

Figure S4. Components of a pixel, including units, a lid, and a base.

1.2.3 Measured force-displacement curves of units

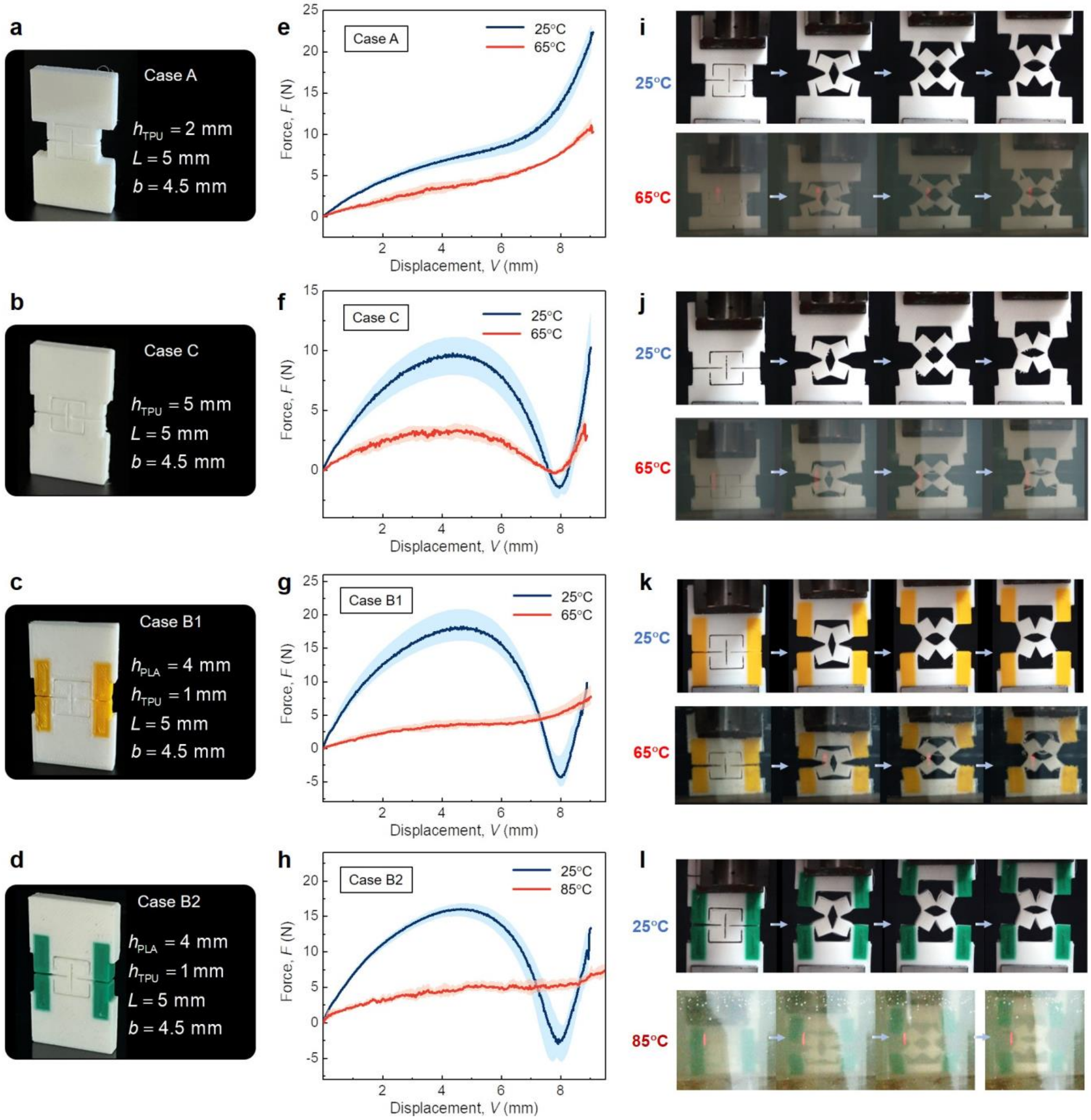

Figure S5. Tensile tests of units at 25°C, 65°C, and 85°C. a-d) Geometrical dimensions of Cases A, B1, B2, and C. e-h) Experimentally measured force-displacement behaviors of the units at different temperatures. i-l) Images of experimentally observed stretching process. (a and e) Case A, always monostable. (b and f) Case C, always bistable. (c and g) Case B1, bistable at 25°C and monostable 65°C. (d and h) Case B2, bistable at 25°C and monostable at 85°C.

2. Theoretical model

2.1 Mechanical model of bi-material kirigami-based units

In the main text, bi-material kirigami-based units are stretched, released, and heated in steps, producing the transformation of the unit between different states. The underlying physical mechanism (i.e., tunable bistability) of the unit needs to be investigated quantitatively. First, referring to the previous work[1,2], we establish the equivalent mechanical model for these bi-material units shown in Figure S6.

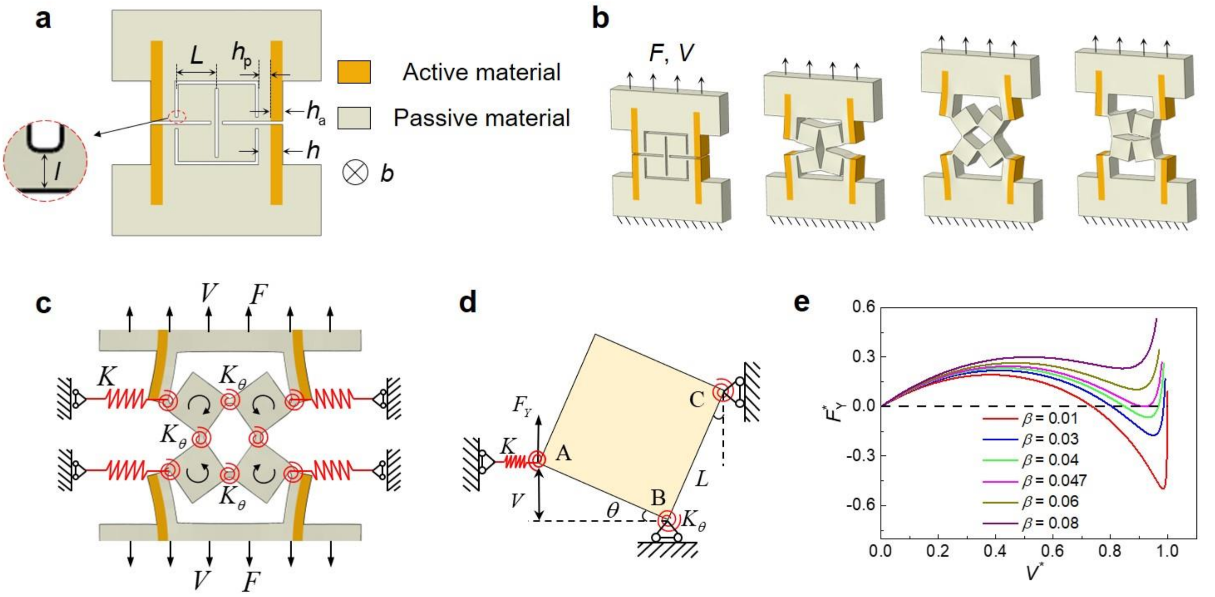

Figure S6. Theoretical model of the bi-material kirigami-based unit. a) Bi-material kirigami-based unit. b) Sequential deformations of the unit. c, d) The deformation pattern of the unit and the equivalent mechanical model. e) Normalized force-displacement responses for various β (e.g., for 0.01 to 0.08).

The bi-material unit (Figure S6a) includes four square plates (edge length L) and four composite cantilevers that have TRPs made of active-material parts (width h_a) and passive-material parts (width h_p). The interconnect ligament between each square and cantilever can be seen as a square with the width of l. The deformation process of the unit upon stretching in the vertical direction is

illustrated in Figure S6b. According to ref. [1], the vertical force F_Y conjugated to displacement V can be written in a dimensionless form as

$$F_Y^* = \frac{F_Y}{KL} = \left(\sqrt{1-V^{*2}} + V^* - 1\right)\left(1 - \frac{V^*}{\sqrt{1-V^{*2}}}\right) + \frac{3\beta \arcsin V^*}{\sqrt{1-V^{*2}}} \quad \text{S1}$$

where $F_Y^* = F_Y/KL$ and $V^* = V/L$ are the non-dimensional force and displacement, and $\beta = K_\theta/\left(KL^2\right)$ is the only parameter in determining the force-displacement response. Figure S6e shows that the critical value of $\beta = 0.047$ can separate bistability and monostability.

2.2 Determination of stiffness *K* and *K*$_\theta$

Then with the help of FEM simulations, we quantify the stiffness of the rotational spring K_θ and the linear spring K in terms of the geometrical parameters l, h_a, h_p, L, and b, and the material parameters of the unit (i.e., Young's moduli E_a and E_p).

First, consider the rotational spring stiffness K_θ. Given the ref.[1], through dimensional analysis, K_θ can be assumed to have the following form of

$$K_\theta = \alpha_1 E_p b l^2 \quad \text{S2}$$

in which α_1 is a dimensionless coefficient to be determined. Through FEM simulations of interconnected ligaments with different sizes, it can be obtained as $\alpha_1 = 0.08$.

Second, consider the linear spring stiffness K. It approximates the effects of the cantilevers with the geometrical parameters h_a, h_p, b, and L defined in Figure S6a. Dimensional analysis indicates

$$K = \alpha_2 \frac{E'I'}{L^3} \quad \text{S3}$$

where α_2 is the dimensionless coefficient and $E'I'$ is the equivalent flexural rigidity of the composite cantilever. And $E'I'$ can be obtained as

$$E'I' = \frac{bE_\mathrm{a}E_\mathrm{p}h_\mathrm{a}h_\mathrm{p}(h_\mathrm{a}+h_\mathrm{p})^2}{4(E_\mathrm{a}h_\mathrm{a}+E_\mathrm{p}h_\mathrm{p})} + \frac{b}{12}(E_\mathrm{a}h_\mathrm{a}^3 + E_\mathrm{p}h_\mathrm{p}^3). \tag{S4}$$

Combining Eqs. (3) and (4), the linear spring stiffness K can be expressed as

$$K = \alpha_2\left[\frac{bE_\mathrm{a}E_\mathrm{p}h_\mathrm{a}h_\mathrm{p}(h_\mathrm{a}+h_\mathrm{p})^2}{4(E_\mathrm{a}h_\mathrm{a}+E_\mathrm{p}h_\mathrm{p})} + \frac{b}{12}(E_\mathrm{a}h_\mathrm{a}^3 + E_\mathrm{p}h_\mathrm{p}^3)\right]. \tag{S5}$$

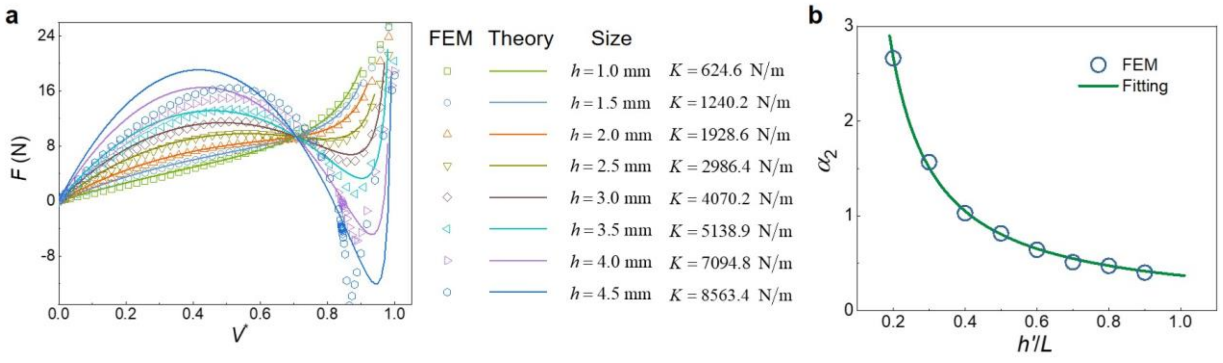

Figure S7. FEM calculated dependence of stiffness K on the geometrical parameters.

In order to evaluate K, we compute the force-displacement responses of the unit with various geometrical parameters using FEM. TPU-made units with different widths of the cantilever are simulated, obtaining the force-displacement curves denoted by symbols in Figure S7a. By fitting the FEM results with the simplified model Equation S1 in conjunction with K_θ from Equation S2, the corresponding values of K can be obtained. Then, the coefficient α_2 can be determined. Therefore, the dependence of α_2 upon h'/L can be quantified and is shown in Figure S7b, in which h' denotes the equivalent width and can be expressed as $h' = \left[\left(E_\mathrm{a}/E_\mathrm{p}\right)^{1/3} h_\mathrm{a}\right]/L + h_\mathrm{p}/L$. Based on the FEM results, a semi-empirical formula for α_2 can be obtained as

$$\alpha_2 = \frac{0.346}{\left(E_\mathrm{a}/E_\mathrm{p}\right)^{1/3}\frac{h_\mathrm{a}}{L}+\frac{h_\mathrm{p}}{L}-0.071}. \quad \text{S6}$$

Through the above analysis, we have established a quantitative relationship between the stiffnesses (K and K_θ) and geometrical parameters (l, h_a, h_p, L, and b).

2.3 Stability phase diagrams of the units

Once K and K_θ are determined, β can be expressed as

$$\beta = \frac{K_\theta}{KL^2} = \frac{2.772\left(l/L\right)^2\left[\left(E_\mathrm{a}/E_\mathrm{p}\right)^{1/3}\frac{h_\mathrm{a}}{L}+\frac{h_\mathrm{p}}{L}-0.071\right]}{\frac{3\frac{h_\mathrm{a}}{L}\frac{h_\mathrm{p}}{L}\left(\frac{h_\mathrm{a}}{L}+\frac{h_\mathrm{p}}{L}\right)^2}{\left(\frac{h_\mathrm{a}}{L}+\frac{E_\mathrm{p}}{E_\mathrm{a}}\frac{h_\mathrm{p}}{L}\right)}+\left[\frac{E_\mathrm{a}}{E_\mathrm{p}}\left(\frac{h_\mathrm{a}}{L}\right)^3+\left(\frac{h_\mathrm{p}}{L}\right)^3\right]}. \quad \text{S7}$$

Equation S7 shows β as a function of four dimensionless parameters, i.e., l/L, $E_\mathrm{a}/E_\mathrm{p}$, h_a/L, and h_p/L. In the following discussion, we fix $l/L = 0.1$ and vary $E_\mathrm{a}/E_\mathrm{p}$ according to the different constituent materials and applied temperatures. With Equation S7, the phase diagram of β_PLA^{25} at 25°C in the $h_\mathrm{TPU}/L - h_\mathrm{PLA}/L$ space is shown in Figure S8a. The green solid line given by $\beta_\mathrm{PLA}^{25} = 0.047$ indicates the bistable-monostable transition boundary. The area in the upper right represents bistability while the lower left regime denotes monostability. When the temperature increases to 65°C, Young's moduli have changed significantly, becoming $E_\mathrm{PLA}^{65} = 5.5\ \mathrm{MPa}$ and $E_\mathrm{TPU}^{65} = 45\ \mathrm{MPa}$. Similarly, the phase diagram of β_PLA^{65} at 65°C in the $h_\mathrm{TPU}/L - h_\mathrm{PLA}/L$ space is shown in Figure S8b, in which the pink solid line given by $\beta_\mathrm{PLA}^{65} = 0.047$ divides it into two parts (i.e., monostable and bistable). Then we put the two boundary lines in Figure S8c to get the range of geometric parameters of units that can realize bistability at 25°C and monostability at 65°C. The phase diagram can then be divided into three parts by two lines in the $h_\mathrm{TPU}/L - h_\mathrm{PLA}/L$ space, that is, Case A, always

monostable with and without heating; Case B1, realizing bistability-to-monostability transition by heating; Case C, always bistable.

Second, as for the units made of TPU and PETG (active material), Young's moduli of PETG are $E_{\mathrm{PETG}}^{25} = 1045\ \mathrm{MPa}$ at 25°C and $E_{\mathrm{PETG}}^{85} = 2.9\ \mathrm{MPa}$ at 85°C. Similar to Figure S8a to c, we can obtain the phase diagrams of $\beta_{\mathrm{PETG}}^{25}$, $\beta_{\mathrm{PETG}}^{85}$, and $\beta_{\mathrm{PETG}}^{25}/\beta_{\mathrm{PETG}}^{85}$ in the $h_{\mathrm{PETG}}/L - h_{\mathrm{PETG}}/L$ space, Figure S8d to f.

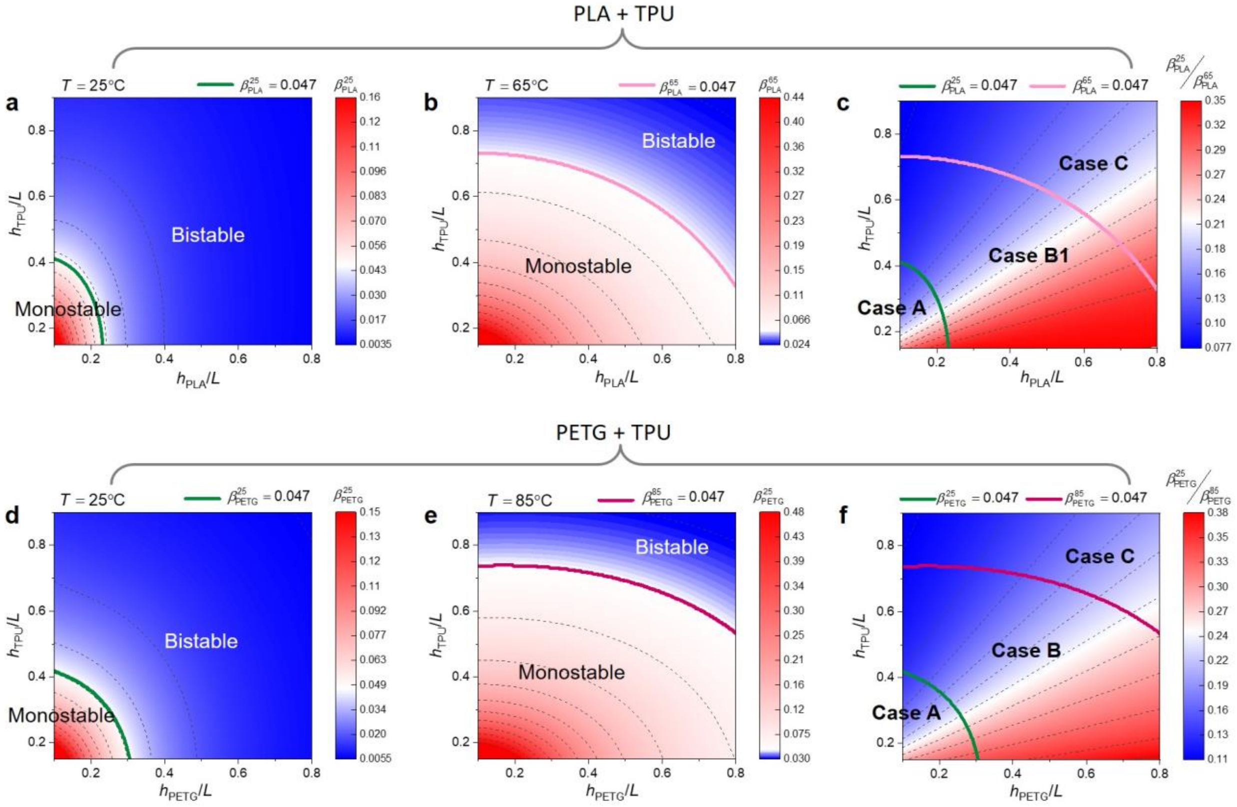

Figure S8. Bistable phase diagram of the units made of (a-c) PLA and TPU, and (D to F) PETG and TPU at different temperatures. a, b) Phase diagrams and contour maps of $\beta_{\mathrm{PLA}}^{25}$ and $\beta_{\mathrm{PLA}}^{65}$ in the $h_{\mathrm{TPU}}/L - h_{\mathrm{PLA}}/L$ space at 25°C and 65°C. Green and pink lines indicate the bistable-monostable transition boundary at 25°C and 65°C for the unit cell made of PLA and TPU. c) Phase diagram for the factor $\beta_{\mathrm{PLA}}^{25}/\beta_{\mathrm{PLA}}^{65}$ in the $h_{\mathrm{TPU}}/L - h_{\mathrm{PLA}}/L$ space. Two lines divide the space into three parts, representing three kinds of unit cells (i.e., Cases A, B, and C) respectively. d-f) Phase diagrams and contour maps of the units made of PETG and TPU at the temperature of 25°C and 85°C.

3. Supplemental results & figures

3.1 Construction of tree diagram

The tree diagrams in Figures 1B and 5A show that the state changes of the pixels realize independent information for each layer. The principle underlying the tree diagram construction is shown in Figure S9. Note that state transitions between layers can be represented by three types of arrows: red arrows indicate no physical implementation, green arrows mean that the state change is beyond definition, and blue arrow denotes that the state change is available.

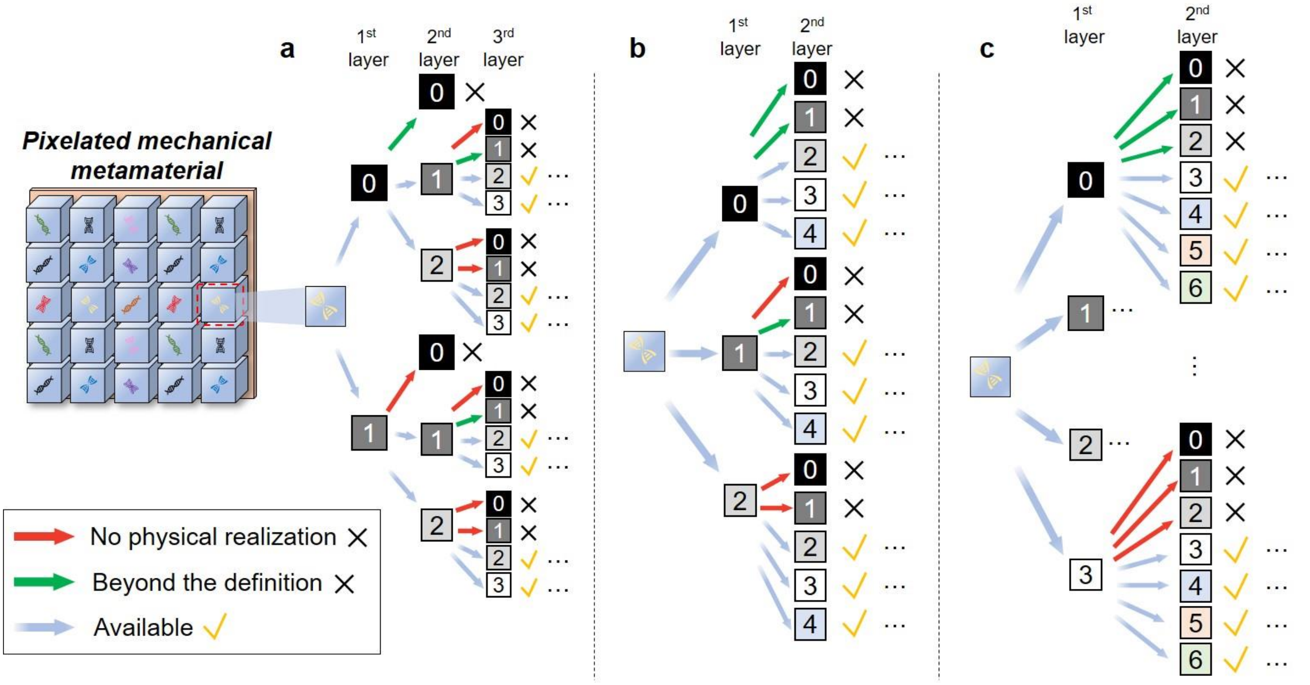

Figure S9. Construction of three diagrams. a) Tree diagram with two states in each layer of information, i.e., ‘0’ and ‘1’ states in the first layer; ‘1’ and ‘2’ states in the second. b) Tree diagram with three available states in each layer. c) Tree diagram with four available states in each layer.

As for the tree diagram (Figure 1b and Figure S9a), each layer of information is expressed by a combination of pixels. In the first layer, states ‘0’ and ‘1’ are selected. We can use the pixels with these two states to construct arbitrary messages in the first layer. As for the second layer of information, the pixels must meet the state changes of ‘0’ to ‘0’, ‘0’ to ‘1’, ‘1’ to ‘0’, and ‘1’ to ‘1’ to achieve independent expression of the second layer of information. However, there is no physical model that can correspond to the state change of ‘1’ to ‘0’ in our work, e.g., the pixel of the kirigami-

based unit cannot achieve ‘1’ to ‘0’ by heating. Therefore, the state transition of ‘1’ to ‘0’ is denoted by a red arrow (Figure S9a). Herein, as an example shown in Figure S10, we encode two layers of information (i.e., ‘T’ for the first and ‘L’ for the second) with Encoder 1-2 that has no ‘1’ to ‘0’ state transition. There is one pixel (colored in green and marked by ‘?’ in Figure S10c) that cannot be built. Therefore, the ‘2’ state is added to the second layer (Figure S9a) so that states ‘1’ and ‘2’ are used to present the information of the second layer. The state change of ‘0’ to ‘0’ is excluded here, denoted by green arrows. As for the third layer of information, the state changes of ‘1’ to ‘1’, ‘1’ to ‘2’, ‘2’ to ‘1’, and ‘2’ to ‘2’ are needed to achieve independent expression of the third layer of information. However, the state change of ‘2’ to ‘1’ cannot be satisfied. Similarly, the ‘3’ state is added into the third layer of information, and together with the ‘2’ state to express the information, realizing state changes of ‘1’ to ‘2’, ‘1’ to ‘3’, ‘2’ to ‘2’, and ‘2’ to ‘3’. As shown in Figure S9a, the state changes of ‘1’ to ‘0’, ‘2’ to ‘0’, and ‘2’ to ‘1’ are deleted and denoted by red arrows, meaning that they cannot be physically realized. The state change of ‘1’ to ‘1’ is denoted by green arrows since it is beyond definition. According to this construction method, the information of the nth layer can be expressed by those of the ‘n-1’ and ‘n’ states.

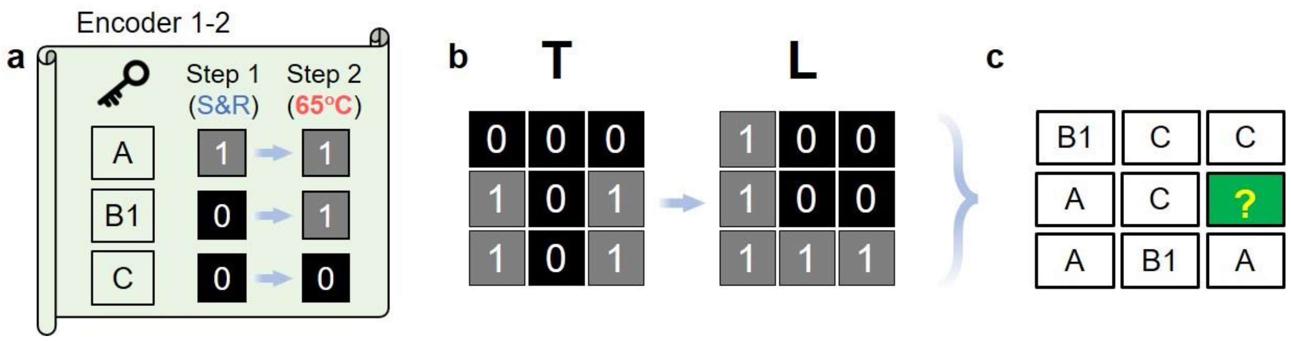

Figure S10. An example of a failed encoding of two layers of information with Encoder 1-2.

As for the tree diagram with three states in each layer of information (Figure 5a, Figure S9b), there are states ‘0’, ‘1’, and ‘2’ in the first layer. Considering that it is not possible to change the states from ‘2’ to ‘0’ and ‘2’ to ‘1’ (marked by red arrows) and the number of states per layer is three, we add states ‘3’ and ‘4’ in the second layer and together with the ‘2’ state to represent the second information. Similarly, the information of the nth layer can be represented in terms of the ‘2n-2’, ‘2n-1’, and ‘2n’ states (Figure 5a).

For the tree diagram with four states in each layer of information, Figure S9c shows that the '0', '1', '2', and '3' states are in the first layer, the '3', '4', '5', and '6' states are in the second layer. Similarly, there are states of '3n-3', '3n-2', '3n-1', and '3n' in the nth layer.

3.2 Finite element method (FEM) simulation

FEM is used to simulate the deformation process of the ciphermaterials, with the commercially available software ABAQUS (version 2017). In the simulations, contact and geometrical nonlinearity are considered. A quasi-static general algorithm is adopted. Note that, to stabilize the simulations, an artificial dissipation factor of 2×10^{-4} is introduced. The model is meshed using hybrid 8-node linear brick elements (i.e., C3D8H elements in ABAQUS) and mesh sensitivity is conducted to ensure numerical convergence. With the properties of materials given in Section 1.1, the materials are modeled as linearly elastic at a given temperature. A multi-step geometrically nonlinear static analysis is performed for stretching loading, unloading, and heating. As for the loading process, displacement-controlled loading is applied to the structure, leading to the snap-through of units. The unloading is realized by removing the load.

3.3 Encoding and storage of two layers of information

The pixelated mechanical metamaterial with two-layer information can be obtained using pixels composed of two units. According to the tree diagram (Figure 1b), four pixels composed of two of Cases A, B1, B2, and C (i.e., A-B1, A-C, B1-B1, B1-C) are shown in Figure S11a to d. By applying a two-step operation (i.e., Step 1, stretch and release; Step 2, heat at 65°C), these pixels can produce the state changes of '1' to '2', '1' to '1', '0' to '2', and '0' to '1'. The encoding approach (Encoder 2-2) of two layers of information is shown in Figure S11e. Combining Encoder 2-2 and discretized pixmaps of 'T' and 'H', design of the corresponding mechanical metamaterial can be obtained (Figure S11f and g). It can be decoded into the two letters (i.e., 'T' and 'H' in Figure S11h) through two operations.

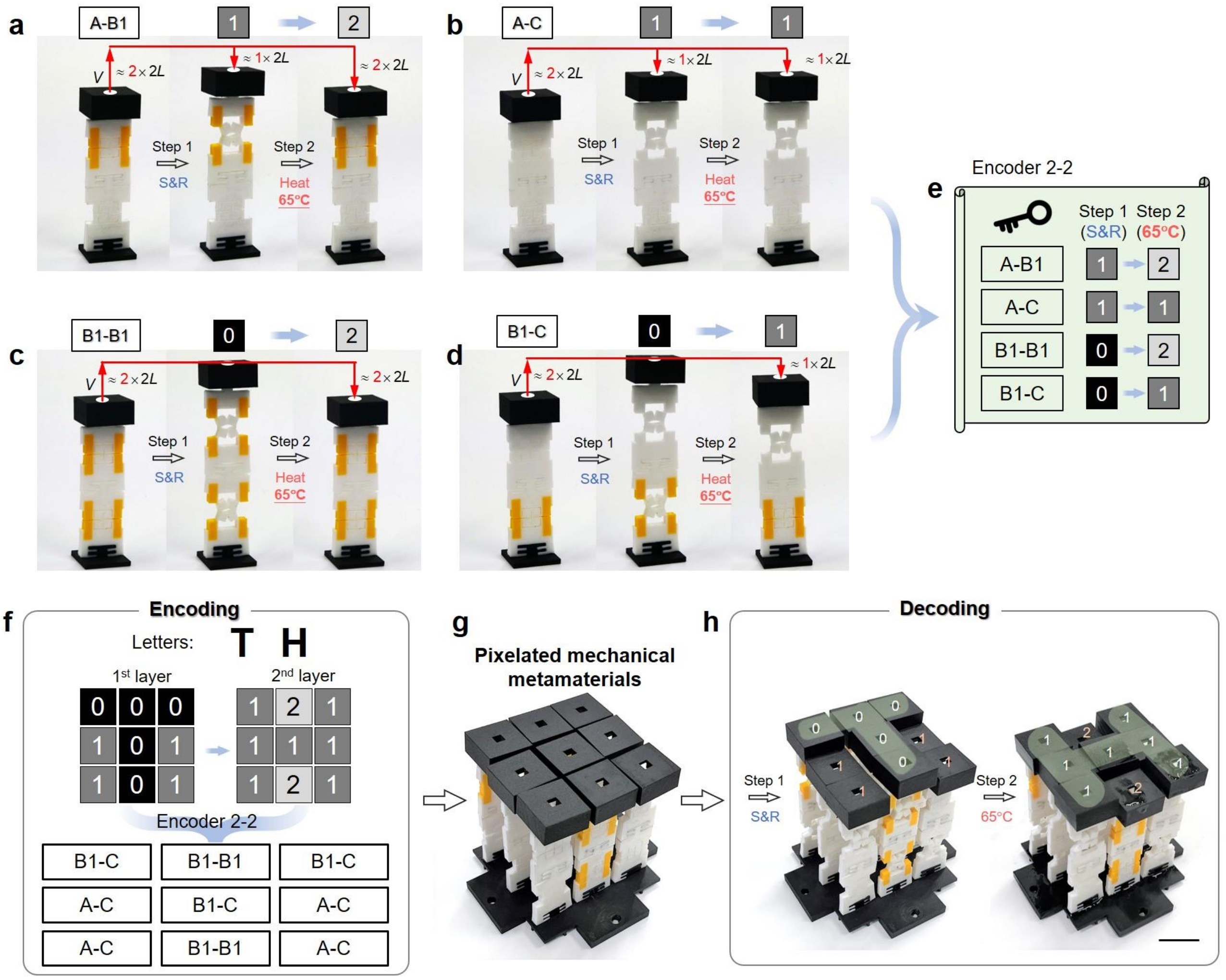

Figure S11. Encoding and storage of two layers information in a pixelated mechanical metamaterial. a-d) Pixels (i.e., A-B1, A-C, B1-B1, B1-C) for four different state changes, i.e., ‘1’→‘2’, ‘1’→‘1’, ‘0’→‘2’, ‘0’→‘1’. e) Two-layer information encoding approach with two states of each layer (Encoder 2-2). f, g) Encoding and storage of the words ‘T H’ in the pixelated mechanical metamaterials with 3×3 pixels. h) Decoding process of the mechanical metamaterial, acquiring the information in turn, i.e., ‘T’→‘U’. Scale bars, 2 cm.

3.4 Matlab-based Encoder

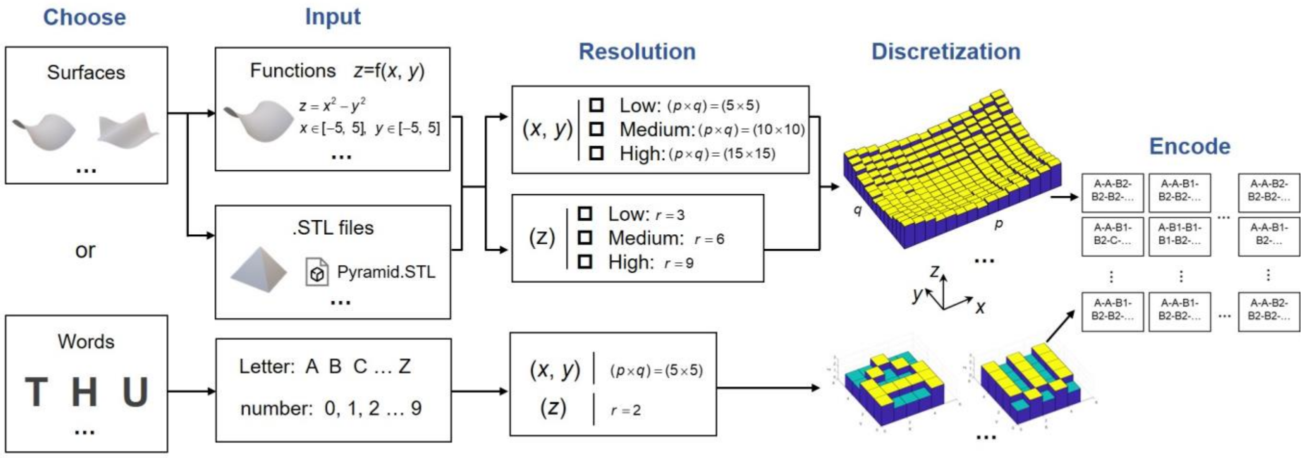

Figure S12. Flow diagram of encoding process for the Matlab-based Encoder.

3.5 Discretization method

A surface fed into the MATLAB GUI is discretized automatically according to the given resolution. The discretization method is shown in Figure S13. As an example, the surface with the expression $z = x$ is given as an input to be discretized into three resolutions (Figure S13a). As for the low resolution, a rectangle mesh is generated for the target surface, producing $5 \times 5 = 25$ grids of the same size. Then, find the centroids of each grid (marked as blue dots) and record their z-coordinate values. The surface is virtually sectioned into three parts along the z direction with the corresponding resolution ($r = 3$) so that the dots that represent the position of grids can fall into one of these sectioned regions. And the dots that fall in the same region are marked with the same color and their corresponding grids are represented by pixels of the same height. Finally, the output approximates the surface by using 25 pixels with three heights. Similarly, increasing the resolution, i.e., increasing the values of p, q, and r, allows the discretized surface to represent the surface more accurately. Moreover, two examples with the expressions $z = xe^{-x^2-y^2}$ and $z = x^2 + y^2$ are discretized in high resolution (see Figure S13b).

As for the discretization of alphabets (A-Z) and numbers (0-9), we use 5×5 pixels to describe them as shown in Figure S14. Herein, two states represented by black and white pixels are adopted to express the information.

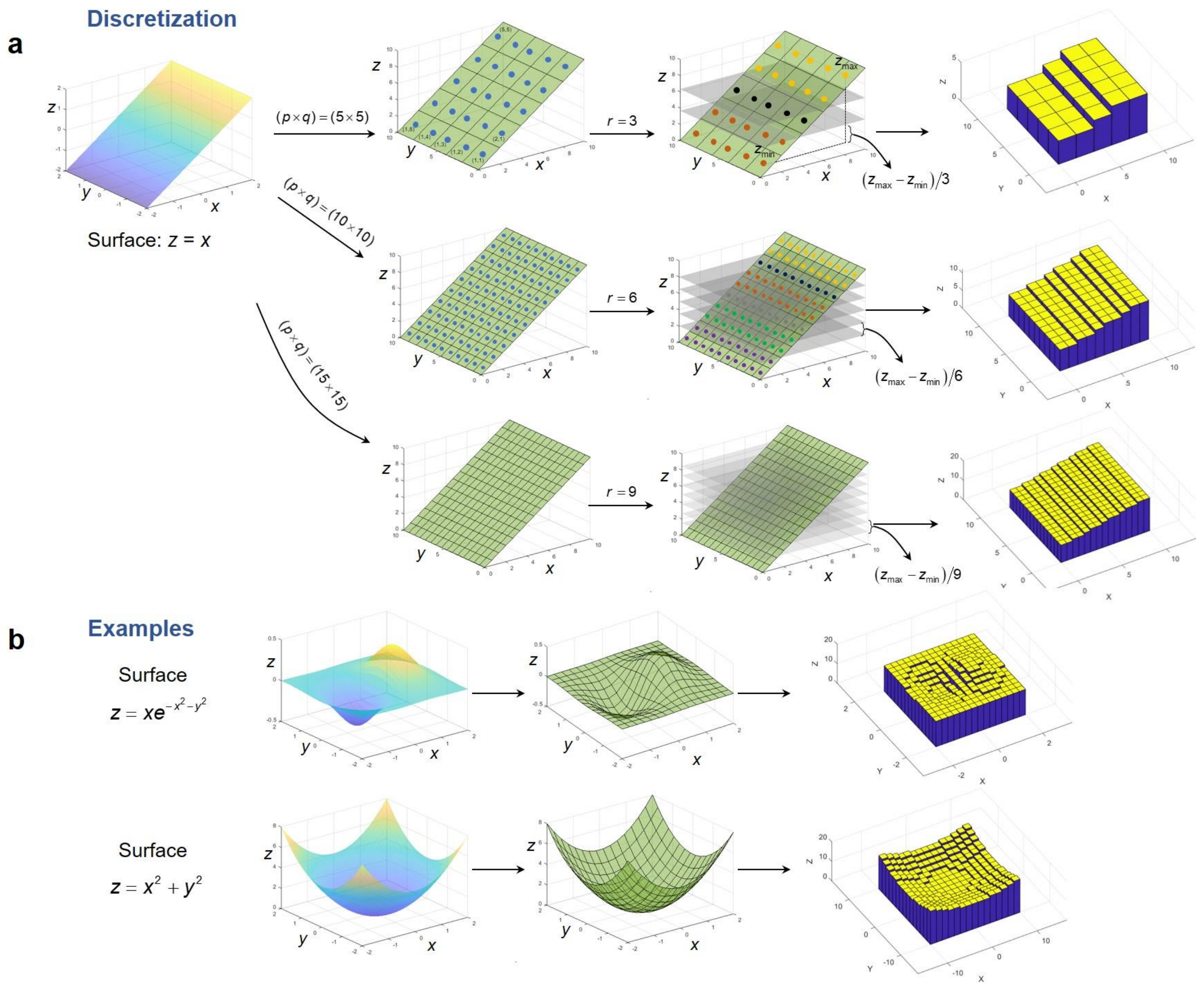

Figure S13. Discretization method of surfaces.

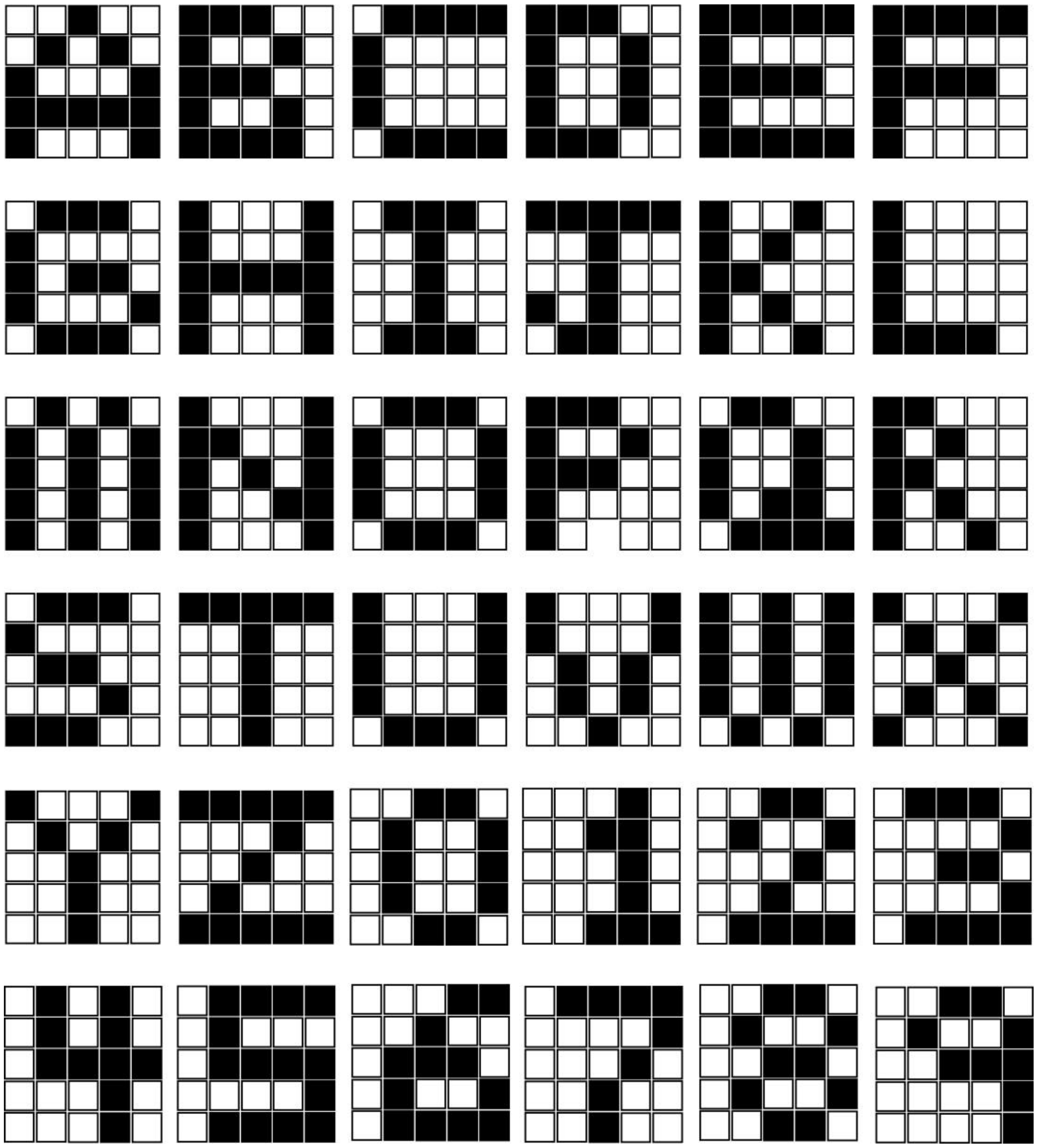

Figure S14. Discretization of letters and numbers.

3.6 Encoding of surfaces with different resolutions

Here, we discretize and encode three surfaces (i.e., pyramid, wave, and hyperbolic paraboloid) with three different resolutions (i.e., low, medium, and high).

------------------ **Low resolution** $(p, q, r) = (5, 5, 3)$ ------------------

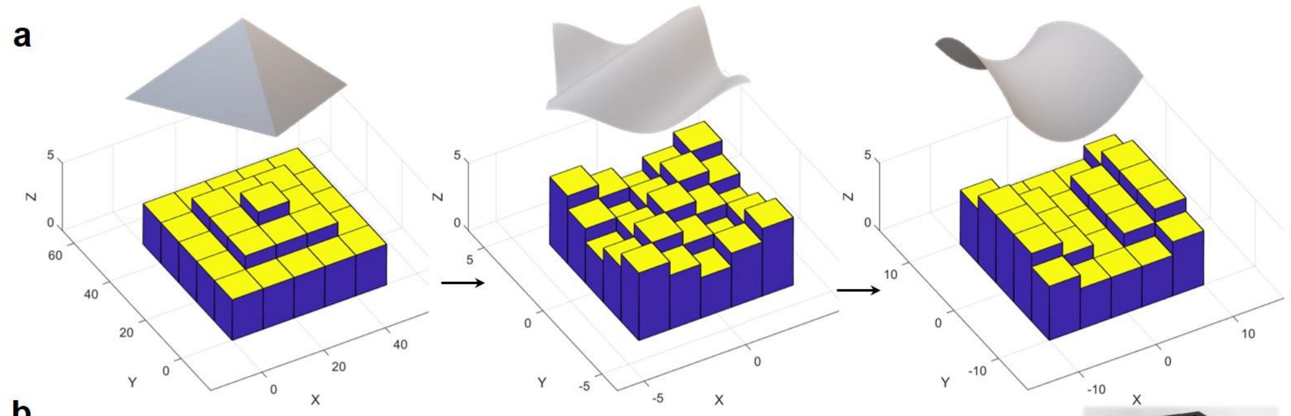

b

A-A-B2-B2-B2-C	A-A-B1-B2-B2-B2	A-A-B1-B1-B2-B2	A-A-B1-B2-B2-B2	A-A-B2-B2-B2-C
A-A-B1-B2-C-C	A-B1-B1-B1-B2-C	A-B1-B1-B2-B2-B2	A-B1-B2-B2-B2-C	A-A-B1-B2-C-C
A-A-B1-B1-C-C	A-B1-B1-B2-B2-C	B1-B1-B2-B2-B2-B2	A-B1-B1-B2-B2-C	A-A-B1-B1-C-C
A-A-B1-B2-C-C	A-B1-B2-B2-B2-C	A-B1-B1-B2-B2-B2	A-B1-B1-B1-B2-C	A-A-B1-B2-C-C
A-A-B2-B2-B2-C	A-A-B1-B2-B2-B2	A-A-B1-B1-B2-B2	A-A-B1-B2-B2-B2	A-A-B2-B2-B2-C

c

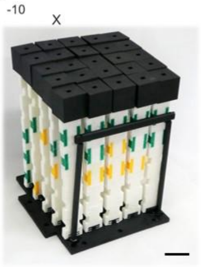

Figure S15. Discretization and encoding of three surfaces at low resolution (i.e., $p = q = 5$, $r = 3$). a) Discretization of three surfaces into 5×5 pixels with three different heights. b) Arrangement of the ciphermaterial encoded with three surfaces. c) The fabricated ciphermaterial.

a ----------- **Medium resolution** $(p, q, r) = (10, 10, 6)$ -------------

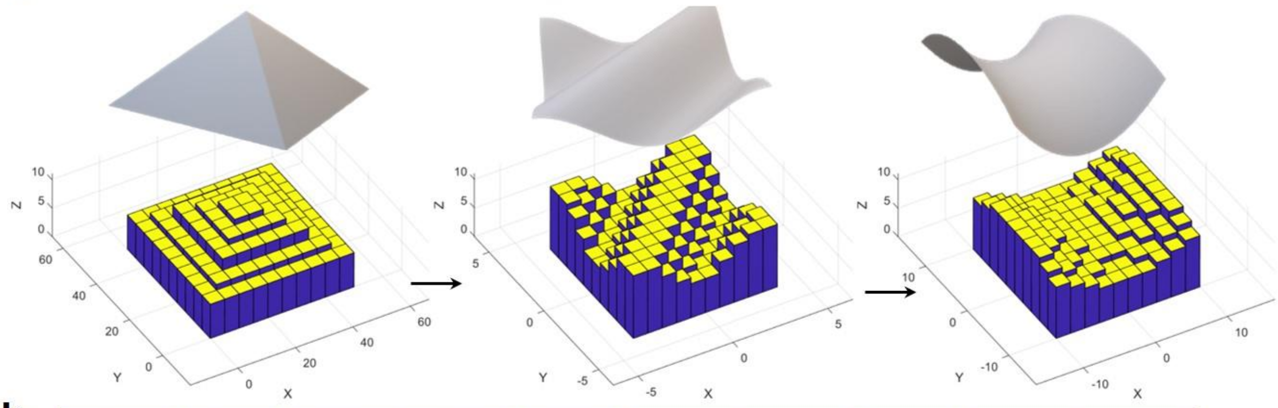

b

5A-0B1-7B2-3C	5A-0B1-8B2-2C	5A-1B1-8B2-1C	5A-3B1-7B2-0C	5A-5B1-5B2-0C	5A-5B1-5B2-0C	5A-4B1-6B2-0C	5A-2B1-7B2-1C	5A-0B1-8B2-2C	5A-0B1-7B2-3C
5A-0B1-6B2-4C	4A-2B1-6B2-3C	4A-4B1-6B2-1C	4A-6B1-4B2-1C	4A-6B1-5B2-0C	4A-5B1-6B2-0C	4A-3B1-7B2-1C	4A-1B1-9B2-1C	4A-1B1-7B2-3C	5A-0B1-6B2-4C
5A-1B1-4B2-5C	4A-4B1-4B2-3C	2A-8B1-3B2-2C	2A-8B1-4B2-1C	2A-7B1-5B2-1C	2A-5B1-7B2-1C	2A-3B1-9B2-1C	2A-3B1-8B2-2C	4A-1B1-7B2-3C	5A-2B1-3B2-5C
5A-3B1-2B2-5C	4A-6B1-1B2-4C	2A-8B1-3B2-2C	1A-8B1-4B2-2C	1A-6B1-7B2-1C	1A-4B1-9B2-1C	1A-4B1-8B2-2C	2A-3B1-8B2-2C	4A-3B1-4B2-4C	5A-4B1-1B2-5C
5A-5B1-0B2-5C	4A-6B1-1B2-4C	2A-7B1-3B2-3C	1A-6B1-6B2-2C	0A-5B1-9B2-1C	0A-5B1-9B2-1C	1A-4B1-8B2-2C	2A-5B1-5B2-3C	4A-5B1-2B2-4C	5A-5B1-0B2-5C
5A-5B1-0B2-5C	4A-5B1-2B2-4C	2A-5B1-5B2-3C	1A-4B1-8B2-2C	0A-5B1-9B2-1C	0A-5B1-9B2-1C	1A-6B1-6B2-2C	2A-7B1-3B2-3C	4A-6B1-1B2-4C	5A-5B1-0B2-5C
5A-4B1-1B2-5C	4A-3B1-4B2-4C	2A-3B1-8B2-2C	1A-4B1-8B2-2C	1A-4B1-9B2-1C	1A-6B1-7B2-1C	1A-8B1-4B2-2C	2A-8B1-3B2-2C	4A-6B1-1B2-4C	5A-3B1-2B2-5C
5A-2B1-3B2-5C	4A-1B1-7B2-3C	2A-3B1-8B2-2C	2A-3B1-9B2-1C	2A-5B1-7B2-1C	2A-7B1-5B2-1C	2A-8B1-4B2-1C	2A-8B1-3B2-2C	4A-4B1-4B2-3C	5A-1B1-4B2-5C
5A-0B1-6B2-4C	4A-1B1-7B2-3C	4A-1B1-9B2-1C	4A-3B1-7B2-1C	4A-5B1-6B2-0C	4A-6B1-5B2-0C	4A-6B1-4B2-1C	4A-4B1-6B2-1C	4A-2B1-6B2-3C	5A-0B1-6B2-4C
5A-0B1-7B2-3C	5A-0B1-8B2-2C	5A-2B1-7B2-1C	5A-4B1-6B2-0C	5A-5B1-5B2-0C	5A-5B1-5B2-0C	5A-3B1-7B2-0C	5A-1B1-8B2-1C	5A-0B1-8B2-2C	5A-0B1-7B2-3C

Figure S16. Discretization and encoding of three surfaces at medium resolution (i.e., $p = q = 10$, $r = 6$). a) Discretization of three surfaces into 10×10 pixels with six different heights. b) Arrangement of the pixelated mechanical metamaterial encoded with three surfaces.

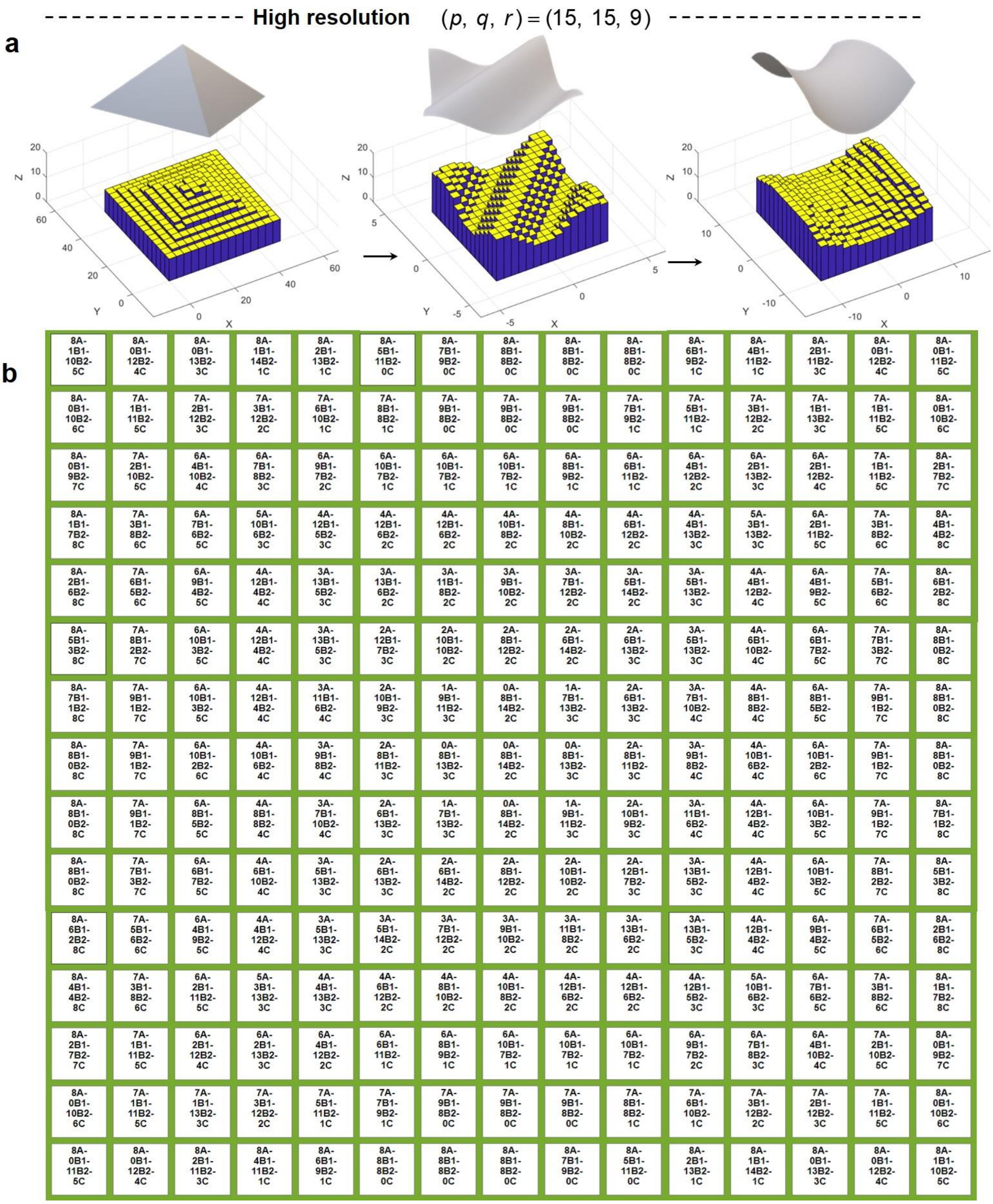

Figure S17. Discretization and encoding of three surfaces at high resolution (i.e., $p = q = 15,\ r = 9$). a) Discretization of three surfaces into 15×15 pixels with nine different heights. b) Arrangement of the pixelated mechanical metamaterial encoded with three surfaces.

3.7 Kirigami-based bimaterial units with different TRPs

To tailor the temporal responses of the units by thermal management, four units with different porosities are fabricated by 3D printing (Figure S18). The cross-section of the TRPs is a hollow square. Three differently sizes with dimensions $5.0\ \mathrm{mm} \times 4.5\ \mathrm{mm}$ and thickness of $0.5\ \mathrm{mm}$, $1.0\ \mathrm{mm}$, and $1.5\ \mathrm{mm}$ are considered. The porosities of TRPs are 0.58, 0.28, 0.08, and 0.

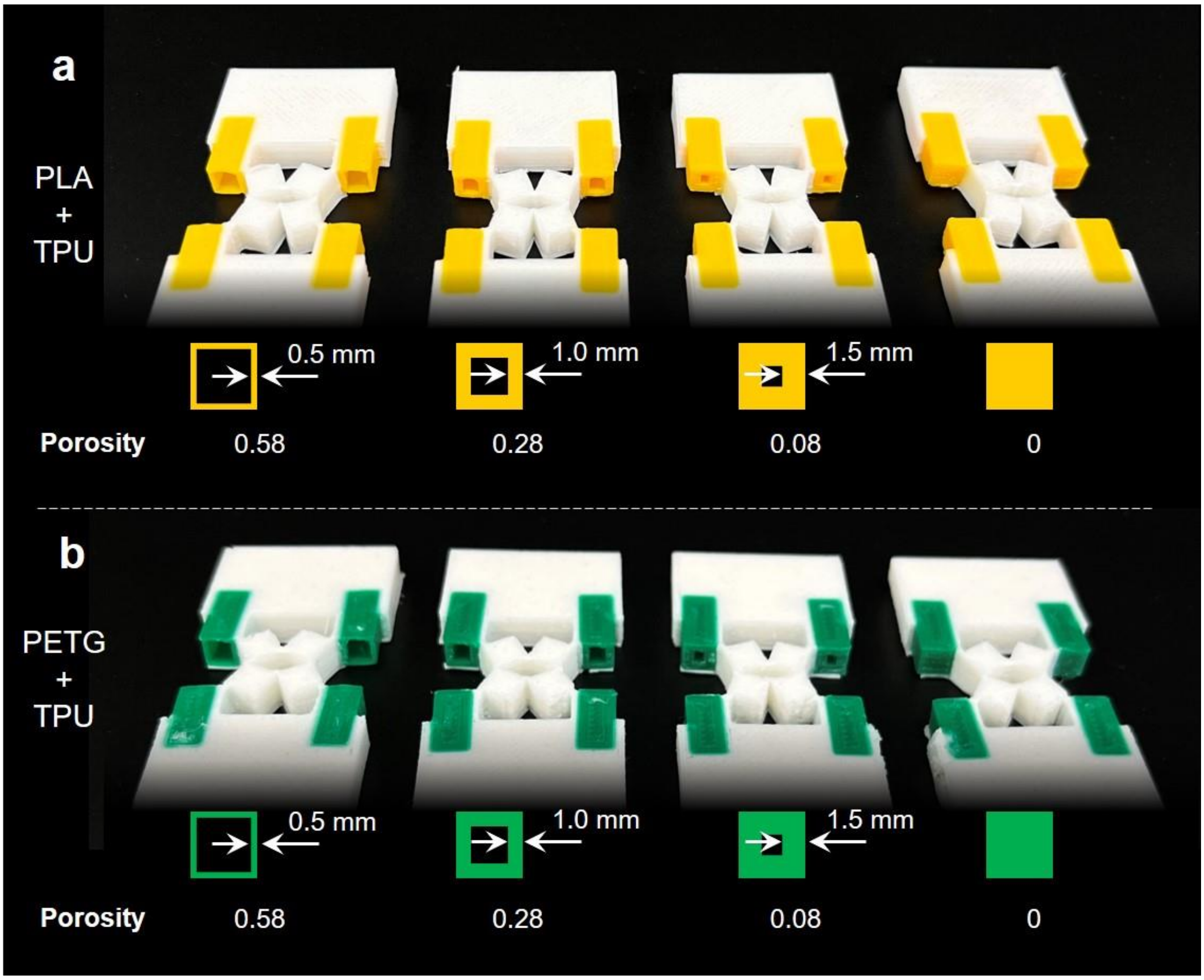

Figure S18. Kirigami-based bi-material units with different TRPs.

3.8 Additional encoding approach

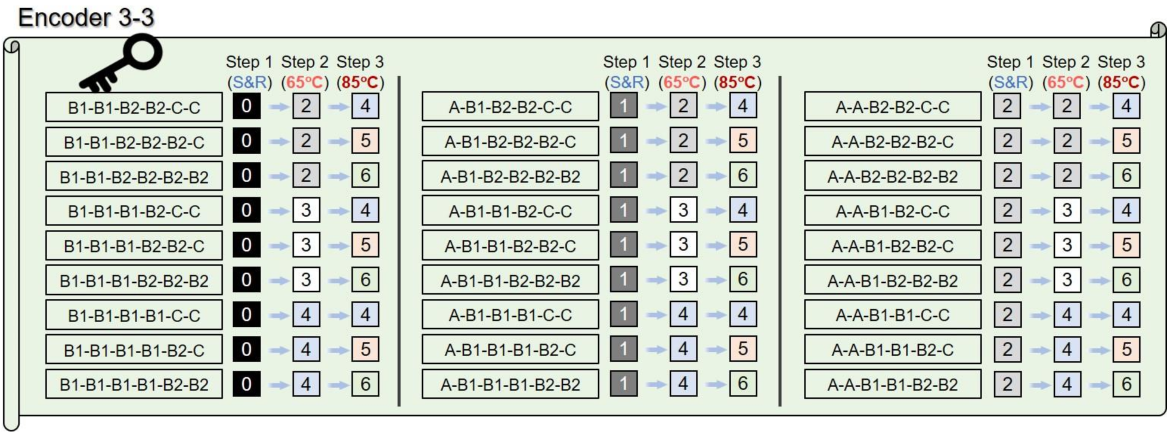

Figure S19. Three-layer information encoding approach (Encoder 3-3) with three states for each layer.

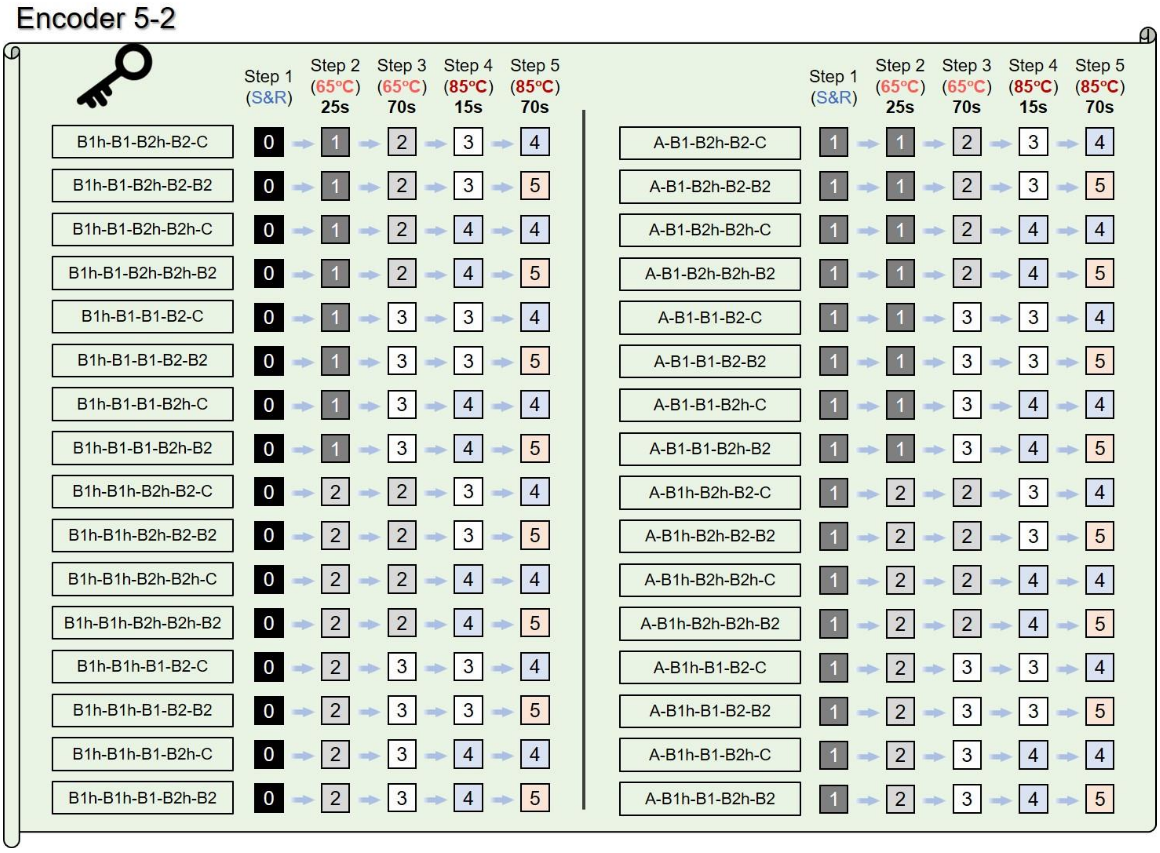

Figure S20. Five-layer information encoding approach (Encoder 5-2) with two states for each layer. The loading sequence is as follows: Step 1, stretch and release (S&R); Step 2, heat in the water at 65°C for 25s; Step 3, heat in the water at 65°C for 70s; Step 4, heat at 85°C for 15s; Step 5, heat at 85°C for 70s.

4. Captions for Videos

Video S1: Design and mechanical responses of pixels.

Video S2: Encoding and storage of one layer of information in a pixelated mechanical metamaterial. The first example is erasable information encoding. The second is heating-visible information encoding.

Video S3: Encoding and storage of two layers of information in a pixelated mechanical metamaterial. Here, the pixelated mechanical metamaterial with two-unit-constructed pixels achieves the encoding and storage of two layers of information, i.e., ‘T’ for the first, ‘H’ for the second.

Video S4: Encoding and storage of one layer of information in a pixelated mechanical metamaterial. Here, the pixelated mechanical metamaterial with three-unit-constructed pixels achieves the encoding and storage of three layers of information, i.e., ‘T’ for the first, ‘H’ for the second, and ‘U’ for the third.

Video S5: Encoding and storage of three layers of 3D surface information in a pixelated mechanical metamaterial. Here, three surfaces (i.e., pyramid, wave, and hyperbolic paraboloid) are encoded into the pixelated mechanical metamaterial. Through three-step operation (i.e., stretching and releasing, 65°C heating, and 85°C heating), the information can be displayed.

Video S6: MATLAB-based Encoder. Here, a Matlab-based tool is provided to enable the design of the pixelated mechanical metamaterial so that the desired arbitrary information can be encoded and stored.

Video S7: Tailored temporal responses by thermal management. Bi-material printed units of Case B1h and Case B2h with hollow TRPs are introduced to fabricate pixels. Besides the regulation through temperatures, time is considered as an extra key factor.

5. References

1. Meng, Z., Chen, W., Mei, T., Lai, Y., Li, Y., and Chen, C.Q. (2021). Bistability-based foldable origami mechanical logic gates. Extreme Mech. Lett. *43*, 101180.

2. Meng, Z., Liu, M., Yan, H., Genin, G.M., and Chen, C.Q. (2022). Deployable mechanical metamaterials with multistep programmable transformation. Sci. Adv. *8*, eabn5460.
